# Design of amino acid- and carbohydrate-based anticancer drugs to inhibit polymerase η

**DOI:** 10.1038/s41598-022-22810-z

**Published:** 2022-11-02

**Authors:** Sepideh Kalhor, Alireza Fattahi

**Affiliations:** Department of Chemistry, Sharif University of Technology, Tehran, Iran

**Keywords:** Medicinal chemistry, Organic chemistry

## Abstract

DNA polymerase η (polη) is of significant value for designing new families of anticancer drugs. This protein takes a role in many stages of the cell cycle, including DNA replication, translesion DNA synthesis, and the repairing process of DNA. According to many studies, a high level of expression of polη in most cases has been associated with low rates of patients' survival, regardless of considering the stage of tumor cells. Thus, the design of new drugs with fewer side effects to inhibit polη in cancerous cells has attracted attention in recent years. This project aims to design and explore the alternative inhibitors for polη, which are based on carbohydrates and amino acids. In terms of physicochemical properties, they are similar to the traditional anticancer drugs such as Cytarabine (cytosine arabinose). These alternative inhibitors are supposed to disrupt the DNA replication process in cancerous cells and prevent the tumor cells from mitosis. These newly designed structures, which are based on natural products, are expected to be non-toxic and to have the same chemotherapeutic impact as the traditional agents. The combinatorial use of quantum mechanics studies and molecular dynamic simulation has enabled us to precisely predict the inhibition mechanism of the newly designed structure, which is based on carbohydrates and amino acids, and compare it with that of the traditional chemotherapeutic drugs such as Cytarabine. Our results suggest that the inhibitors containing the natural building blocks of amino acid and carbohydrate could be considered alternative drugs for Cytarabine to block polη.

## Introduction

**Figure 1:**
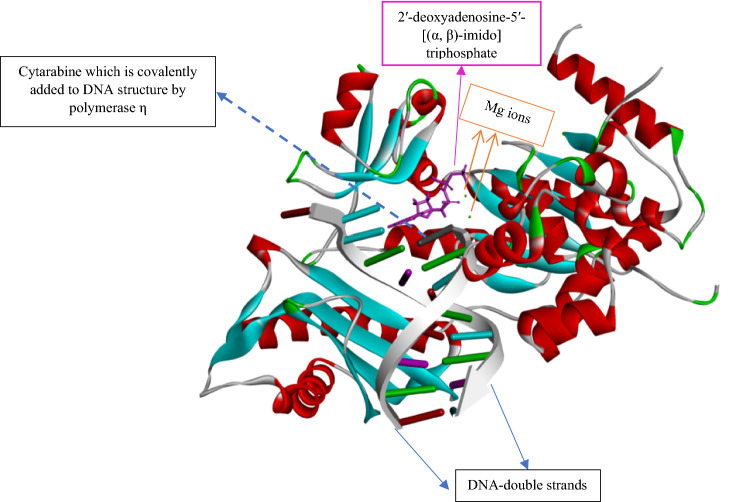
Structure of polymerase η complexed with DNA to which cytarabine is added covalently. Also 2′-deoxyadenosine-5′- [(α, ß)-imido] triphosphate (pink molecule) is incoming nucleotide.

**Figure 2:**
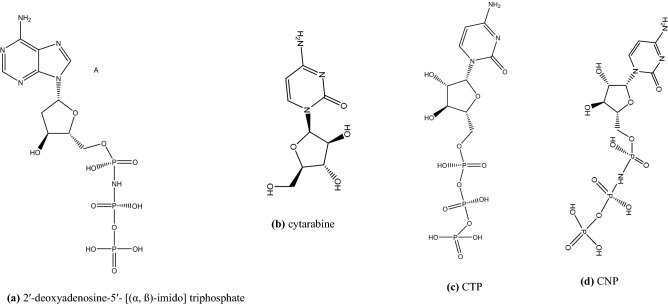
(**a**) 2′-deoxyadenosine-5′- [(α, ß)-imido] triphosphate, (**b**) cytarabine, (**c**) CTP, and (**d**) CNP.

As it is widely known, the correct transmission of chromosomes is valuable for inheritance, which is facilitated by the DNA replication process. The life cycle of cells is not flawless. A variety of environmental agents alongside reactive metabolites generated during the life cycle of cells can cause lesions in DNA structures^1^. On the other hand, neoplastic transformation and genome instability, which are the replication stress results, can be another reason for DNA damage^2^. At first glance, we expect cells to employ pathways to repair DNA damage. Under replication stress, the cells are supposed to replicate the damaged DNA before the mitosis phase of cells' life cycle. Undoubtedly, the copies of this damaged DNA will pass down to both daughter cells, and this mechanism, which is called DNA damage tolerance, will be repeated for daughter cells and result in cancerous cells^3^. Many proteins involved in producing these cancerous cells, including DNA polymerase η, can target the newly designed anticancer drugs. DNA polymerases are participating in crucial steps of DNA replication in that they involve in functioning in the replication of the genomic material, playing a role in DNA repair, and taking part in a process called translesion DNA synthesis (TLS)^4^. Fifteen mammalian DNA polymerases have been identified, but our focus in this study is DNA polymerase η (Polη). Polymerase η among the other family members is a unique enzyme. For instance, it bypasses the barriers caused by dominant UV lesion^5^. According to many studies, a high level of expression of polymerase η has been associated with low rates of patients' survivals^6^. Even in cases such as acute myeloid leukaemia, the polymerase η can be a good target for the design of new anticancer drugs. Many attempts have been made to discover the pathway in which the polymerase η is involved. For example, this enzyme can perform translesion DNA synthesis, even in the presence of cisplatin adducts^7,8^. As the therapeutic drug, Cytarabine is used to treat acute myeloid leukemia and expected to inhibit DNA polymerase η. However, recently, it was found that this drug could form covalent bonds with DNA by the aim of DNA polymerase η^9^. Thus, DNA polymerase η (Polη) can continue DNA synthesis from Cytarabine terminated primers, which leads to drug resistance and DNA mutation. Also, high-resolution structures of the complex of DNA, DNA polymerase η (Polη), and Cytarabine (all three structures together) have been provided (PDB code: 6D0Z)^9^. The above-described issues of Cytarabine attracted our attention to design alternative drugs to inhibit DNA polymerase η (Polη) predominantly. Cytarabine, as the synthetic analog of deoxycytidine, enters the cell in different ways. It can be transported across the cell membrane by a nucleoside carrier system^10^. Diffusion at a high concentration of this drug has been reported, too^11^. Many adverse effects of Cytarabine including myelosuppression^12^, Anaphylactic reactions^13^, Drug Fever^14^, a very painful erythematous palmar-plantar bulla formation^15^, Subacute pulmonary failure^16^, and gastrointestinal toxicity^17^ stimulated our research group to find the natural-based drug that can be used instead of Cytarabine. For 40 years, progress in protein crystallography and NMR has enabled the chemists to have three-dimensional structures of Protein–ligand complexes to visualize the selective interaction of drugs better and to optimize the structures of drugs to achieve more efficient patients' treatment^18^. Simultaneously, the achievements in computational chemistry have allowed scientists to predict molecular interactions and in-silico characterization of protein–ligand complexes^19^. The diversity of carbohydrates in nature has proved to be one of the best tools in drug discovery^20^. Moreover, well-defined stereochemical centers with flexible pyran and furan rings make them functional building blocks to present pharmacophoric groups^21–24^. Also, it has been found that the combination of sugars and peptides or sugars and small molecules can have a specific influence on structural properties such as metabolic stability^25^, solubility, membrane permeability^26^, biodistribution^27^, and ligand-target interactions^28,29^. As a result, our recent interest is to design structures based on sugars and amino acids via computational methods and molecular dynamic (MD) simulations. These designed amino acid-sugar-based structures could be potentially good candidates to replace Cytarabine. We used the structure with the PDB ID of 6D0Z as our initial structure (Fig. 1)^9^. In Fig. 1, the post-insertion step is observed, in which DNA polymerase η has covalently inserted cytarabine residue to one of the DNA strands. Also, in Fig. 1, the small molecule shown by pink color is the incoming nucleotide called 2′-deoxyadenosine-5′-[(α, β)-imido] triphosphate in its inactive form. Green dots in Fig. 1 are Mg^2+^ (to fix the pH of crystallization at 8.0)^9^. Figure 1 also shows the crystallized form of the human Polη catalytic core (residues 1–432) complexed with a DNA including 12-nucleotide template (5′-CATGACAGTGCT-3′)/8-nucleotide primer (5′-AGCACTGT-3′) in which Cytarabine is covalently bonded to the 8-nucleotide strand. Also, as it can be seen in Fig. 1, the human Polη inserts 2′-deoxyadenosine-5′- [(α, ß)-imido] (containing triphosphate) into the DNA structure from the part of DNA terminated with Cytarabine. Thus, we can infer that triphosphate should have been added to Cytarabine before forming the covalent bond with the primer strand of DNA (Fig. 2c). One of the essential steps in the cell cycle is the simultaneous interaction of ((2R,3S,4S,5R)-5-(4-amino-2-oxopyrimidin-1(2H)-yl)-3,4-dihydroxytetrahydrofuran-2-yl) methyl tetrahydrogen (S, S)-triphosphate (CTP) with DNA strands and Polη. We considered this fact to design our new amino acid-sugar-based structure to kill cancerous cells undergoing DNA synthesis by Polη in the S-phase of the cell cycle.

As Cytarabine prodrug (Fig. 2b) is entering the cell, deoxycytidine and pyrimidine kinases will phosphorylate this prodrug and convert it to CTP (Fig. 2c)^30^. As seen in Fig. 2, the structure known as cytarabine-5′-[(α, β)-imido] triphosphate (CNP) (Fig. 2d) is very similar to CTP (Fig. 2c), but CNP is non-hydrolysable compared to CTP. As a result, CNP is expected to be more stable than CTP in the presence of ions such as Mg^2+^ and water molecules. In this study, we have compared our designed structures with CNP. The synthesis of CNP can be achieved by methods described by Hirokazu Nakayama et al.^31^.

Our designed structures are based on amino acids and D-fructose, and their synthesis can be achieved by Amadori and Heyns methods^32^. We assume that our suggested structures can compete with cytarabine-5′- [(α, β)-imido] triphosphate (CNP) in the interactions with Polη and DNA, as mentioned above. In this study, the summary of our approach toward drug design can be explained as follows. First, the suggested structures were optimized at the B3LYP/6-311++G (d, p) level^33^. Then, the docking procedures were performed to predict the conformations and binding affinities of the drugs (complexed with DNA and DNA polymerase η (Polη)) studied herein including CNP and our suggested structures using the Auto Dock/Vina plugin with scripts from the Auto Dock Tools Package^34,35^. The intermolecular interactions, including hydrogen bonds, electrostatic interactions, and van der Waals for these drugs complexed with DNA and DNA polymerase η (Polη) were analyzed by discovery studio modeling environment (Discovery studio 4.5)^36^. Finally, the extracted data from the previous steps were used as inputs for MD simulation (molecular dynamic simulation) (The GROMACS 5.2)^37^. Whereas the experimental techniques are valuable tools to predict the behavior of biomolecular dynamics, some details such as pressure distributions inside membranes cannot be accessible through experiments. To address this issue, in this study, we applied molecular dynamic simulation (parameterized using the experimental data) to explore the interaction of the drugs with DNA and DNA polymerase η (Polη))^38^.

## Method and theoretical calculations

### Molecular docking and Geometry optimizations of the suggested structures

**Figure 3:**
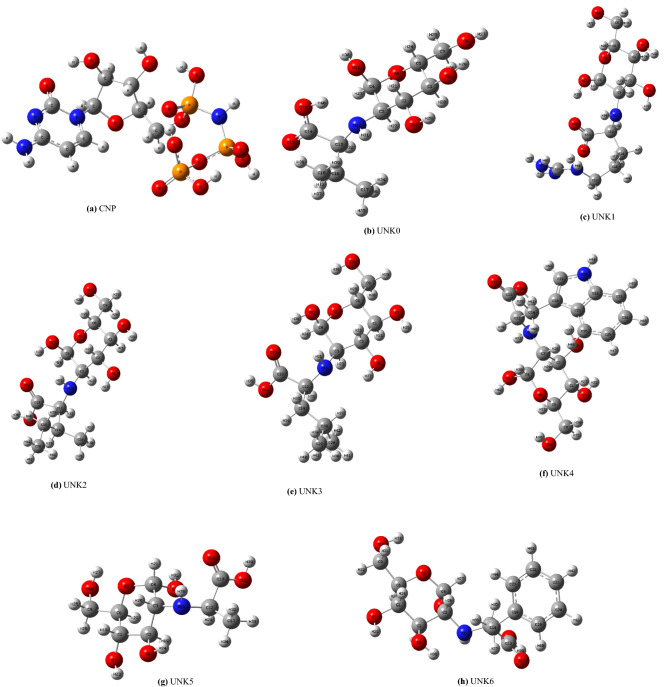
Optimized geometries of the lowest energy conformers of the suggested drugs based on carbohydrate and amino acids at B3LYP/6‐311++G (d, p) level (see Table 1 for nomenclatures and codes of molecules).

The knowledge about the gas phase structural properties (including electronic properties, bond lengths, angles, and proper dihedrals) of CNP and our amino acid-carbohydrate-based suggested structures is necessary before the docking process and molecular dynamic simulations. The initial conformational searches at the relative energy of 10 kcal/mol were carried out with the MMFF force field (molecular mechanics) using Spartan software^33^. The geometry optimizations and frequency calculations were carried out using the 6-311++G** basis set. The absence of imaginary frequencies confirmed that the optimized structures corresponded to the real minima^33^. The optimized structures of CNP and suggested structures are illustrated in Fig. 3. See Table 1 for nomenclatures and codes of molecules. For example, UNK4 refers to ((2S,3R,4R,5S,6R)-2,4,5-trihydroxy-6-(hydroxymethyl) tetrahydro-2H-pyran-3-yl)-L-tryptophan structure (Table 1, entry 6). Before the docking procedure, the initial PDB was edited by VMD package^39^. All water molecules and other solvents, as well as the co-crystallized ligands including nucleotide (2′-deoxyadenosine-5′- [(α, ß)-imido] triphosphate) and Cytarabine, incorporated in DNA, were removed using Discovery studio 4.5. To perform a docking procedure by Auto Dock/Vina, we considered the complex of protein and DNA as our receptors and CNP and designed structures as ligands. Then, the PDB files of the receptors and the ligands were saved as pdbqt files by auto dock tools^40^. In pdbqt files, information such as atomic charges, atom-type definitions, and rotatable bonds (like single bonds) is stored^34^. We used grid box with the dimensions 20 × 24 × 28 Å^3^ with its center located at x = 52.861, y = 6.193, z = − 12.977 (Table 1).

**Table 1:**
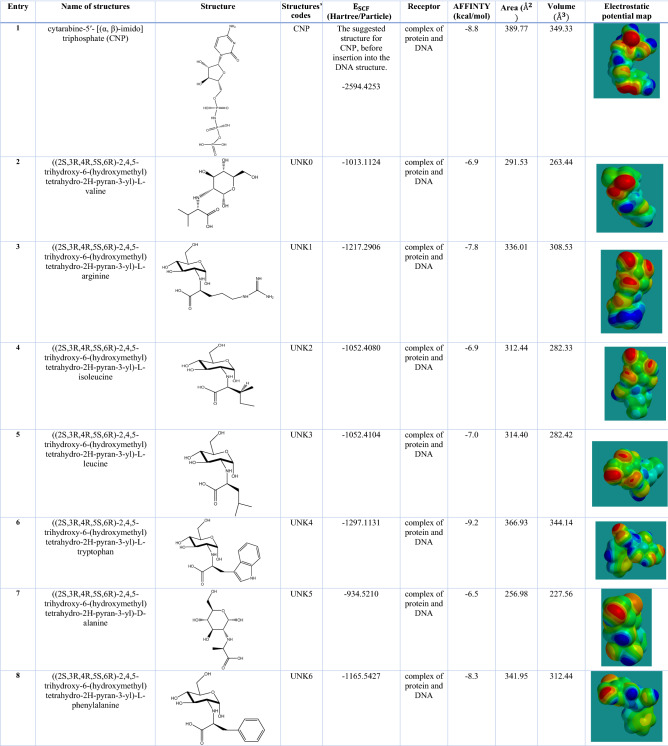
The structural information for cytarabine-5′-triphosphate and our designed amino acid-carbohydrate based structures.

**Figure 4:**
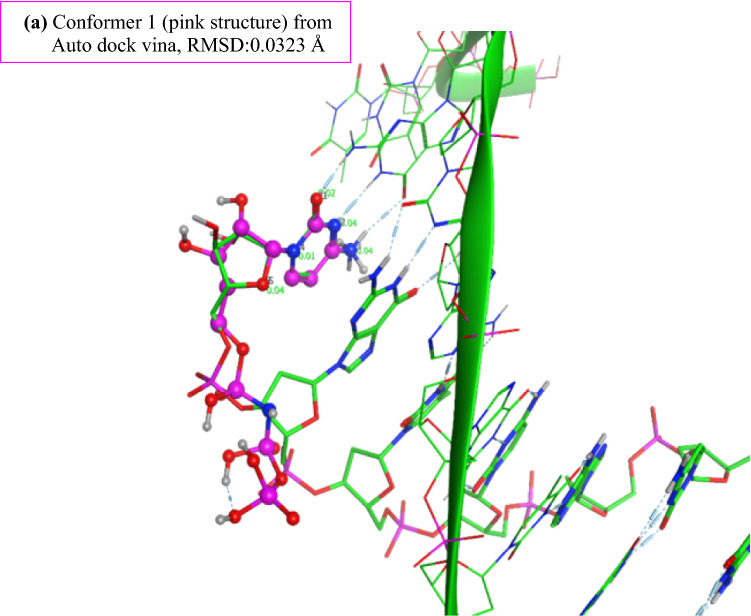
The structural information (obtained by Auto Dock Vina) for the first conformer of CNP (brown) complexed with DNA and DNA polymerase η (Polη) with the least RMSD = 0.0323 Å (structure of Polη not shown here).

To find the best conformer for CNP (which will be used for MD calculations), we used Auto Dock Vina, which suggested nine conformers. Figure 4 illustrates the structural information (obtained by Auto Dock Vina) for the first conformer of CNP complexed with DNA and DNA polymerase η (Polη) with the least RMSD = 0.0323 Å. This RMSD was calculated by overlaying this conformer over the Cytarabine structure obtained from the main pdb file (6D0Z). The structural information for all nine conformers of CNP is given in Fig. S1 in Supporting Information.

As seen in Table 1, our approach to design the new structures as anticancer drugs are based on computing some physical properties such as volume, area, and electrostatic potential map (EPS) of the designed structures, and comparison of these properties with those of the main drug, which is CNP in this study. The volume, area, and electrostatic potential map (EPS) of CNP and designed structures (listed in Table 1) were obtained by single-point energy calculations on the structures optimized at the B3LYP/6‐311++G (d, p) level (in a vacuum) by Spartan software^32^. The last column of Table 1 represents the EPS. The red color depicts the regions with a higher electron density (nucleophilic centers), and blue color depicts the regions with a lower electron density (electrophilic centers). The EPS patterns allow us to select the candidate structures (designed structures) with the charge distribution most similar to the charge distribution of the main drug (i.e., CNP). Comparing the volume, area, EPS, and binding affinity of CNP with those of the designed compounds indicates that UNK4 (Entry 6 in Table 1) should be the most suitable candidate to replace CNP. For instance, in terms of binding affinity obtained by the Auto Dock/Vina, the binding affinity values of our designed structure UNK4 and CNP were calculated to be − 9.2 kcal/mol and − 8.8 kcal/mol, respectively, suggesting that UNK4 could replace CNP.

Moreover, we used Auto Dock Vina to study the interaction of cytarabine (Fig. 2b) and the complex of DNA and DNA polymerase η (Polη). The results from this study can be seen in entry 1 of Table S1. Accordingly, the designed compound UNK4 has a better affinity towards the receptors (including DNA and DNA polymerase η (Polη)) than cytarabine. The binding affinity values of cytarabine (entry 1 of Table S1) and UNK4 (Entry 6 in Table 1) were calculated to be − 6.6 kcal/mol and − 9.2 kcal/mol, respectively, representing that UNK4 can interact more effectively with the complex of DNA and DNA polymerase η than cytarabine.

Also, to explore the probable off-target interactions of the designed compound UNK4, we chose receptors with which cytarabine structure and its derivatives interact. For example, one of the targets was DNA Polymerase λ^41^, having a significant role in meiotic recombination and DNA repair^42^; the interactions of both structures, including cytarabine and UNK4, with Polymerase λ, were evaluated (entries 2 and 3, Table S1). Our designed compound UNK4 and cytarabine binding affinity values towards Polymerase λ were calculated to be − 8.5 kcal/mol and − 7.9 kcal/mol. These results show that UNK4 has more affinities towards receptors, including DNA and DNA polymerase η (Polη) than Polymerase λ. In contrast, cytarabine has more affinity towards Polymerase λ than towards receptors in this study, suggesting that UNK4 can interact better with receptors including DNA and DNA polymerase η (Polη) in comparison with cytarabine.

The other target was Human deoxyribonucleoside kinase playing a valuable role in the phosphorylation of the natural deoxyribonucleosides^43^. Also, this enzyme phosphorylates a significant number of nucleoside-based drugs^43^, which are vastly used as anticancer drugs such as cytarabine. As a result, we performed a docking process for cytarabine and dCK. Simultaneously, the binding affinity of UNK4 and dCK was obtained by the Auto Dock/Vina. The results can be observed in entries 4 and 5, Table S1. According to entries 4 and 5, Table S1, UNK4 and cytarabine bear binding affinity towards dCK as − 7.2 kcal/mol and − 7.7 kcal/mol, showing that UNK4 can possibly interact more effectively with receptors, including DNA and DNA polymerase η (Polη) compared to dCK. The studies about the possible mentioned off-targets interactions of UNK4 alongside with the results from Table 1 show that UNK4 can be a good replacement for cytarabine.

### Prediction of log P for the designed anticancer structure

P is the partition coefficient defined as the ratio of the concentration of neutral drug molecule in the lipophilic solvent to its concentration in water. The lipophilicity and hydrophilicity of drug molecules are given by the logarithm of P (log P). 1-octanol is usually considered the lipophilic solvent because it represents properties similar to the membrane of cells^44^. To illustrate that our designed structure (UNK4) has good solubility in both water and lipophilic solvents, we calculated log P of UNK4. The free energy changes of transferring process of UNK4 from water to 1-octanol can be related to log P of UNK4 using the Eqs. (1) and (2).

1 $$ \Delta {\text{G}}_{{\text{transferring process}}} = \Delta {\text{G}}_{{1}} - \, \Delta {\text{G}}_{{2}} $$

2 $$ \Delta {\text{G}}_{{\text{transferring process}}} = - \;\left( {{\text{RT}}} \right){\text{Ln P}} = - \;{2}.{3}0{3}\left( {{\text{RT}}} \right){\text{ log P}} $$

The free energy changes of transferring UNK4 in both water and 1-octanol solvents were calculated (ΔG_1_ and ΔG_2_, respectively). From the difference between these two free energy changes (ΔG_transferring process_), it is possible to obtain log P^45^. In the quation 2, R is the ideal gas constant, T is temperature (298 K), and P is the partition coefficient.

**Figure 5:**
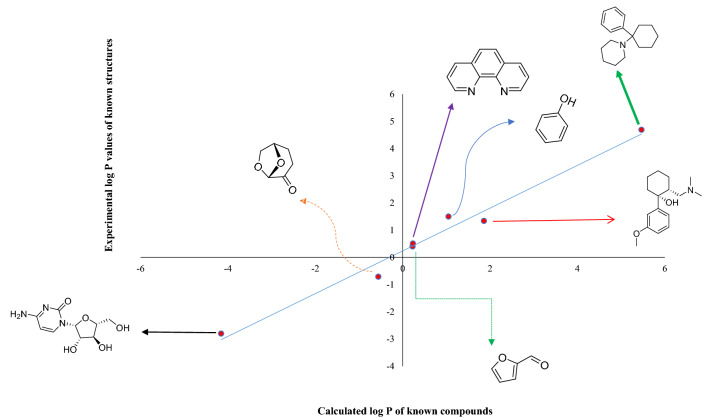
Plot of the experimental log P against the calculated log P (clog P).

First, we computed log P of the structures with known experimentally-measured log P values, including phenol, furfural, dihydrolevoglucosenone^46^, and some known drugs such as Phenanthroline^47,48^, phencyclidine^49^, tramadol^50^ and cytarabine^51^ by employing density functional theory (DFT) calculations in combination with a solvation model based on density (SMD) (refer to Table 2)^52^. Then, we plotted the experimentally reported log P of known structures versus their computed log P (Fig. 5). We used this plot to predict log P of UNK4, which will be discussed below. The geometries of all molecules used in this part of the study were optimized at the M06-2X/6‐311+G (d, p) level^53^ in both water and 1-Octanol solvents^32^. Figures S2 and S3 show the optimized molecules and their charge distributions in water and 1-octanol, respectively. These figures illustrate that the overall charge distribution of atoms in molecules can be different in various solvents. According to the Hessian analysis, no imaginary frequencies were found for the structures, indicating that all of the optimized structures were real minima. The properties of the solvents, such as the dielectric constant, refractive index, bulk surface tension, acidity, and basicity are required to calculate the free energy of each solute in both water and 1-Octanol solvents^46^. Figure 5 represents the plot of the experimentally measured log P values versus clog P (calculated log P) for the compounds listed in the entries 1–7 in Table 2 with $y=0.785-0.241$ $\mathrm{and} {R}^{2 }=0.98$. Where *y* and *x* indicate the experimental and calculated log P values, respectively. This plot predicts the experimental log P value of UNK4 to be − 1.64 and comparing it with the log P value of Cytarabine (− 2.8)^51^ suggests that UNK4 tend to have more distribution in the octanol phase than water, which can have positive effects on the penetration of UNK4 through the cell membrane. In contrast, the log P value of Cytarabine indicates that it is a more hydrophilic molecule.

**Table 2:**
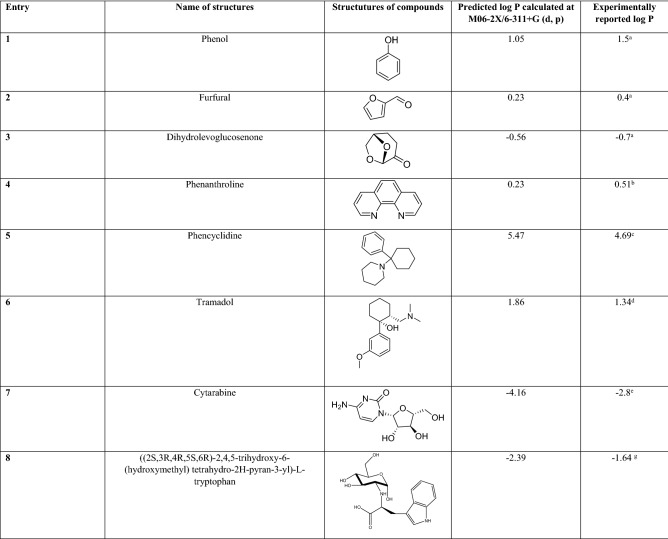
The calculated (at the M06-2X/6‐311+G (d, p) level) and experimental log P values.

References for a-g: a^46^; b^47,48^; c^49^; d^50^; e^51^, g; predicted based on the plot in Fig. 5.

### Grid box searching

**Figure 6:**
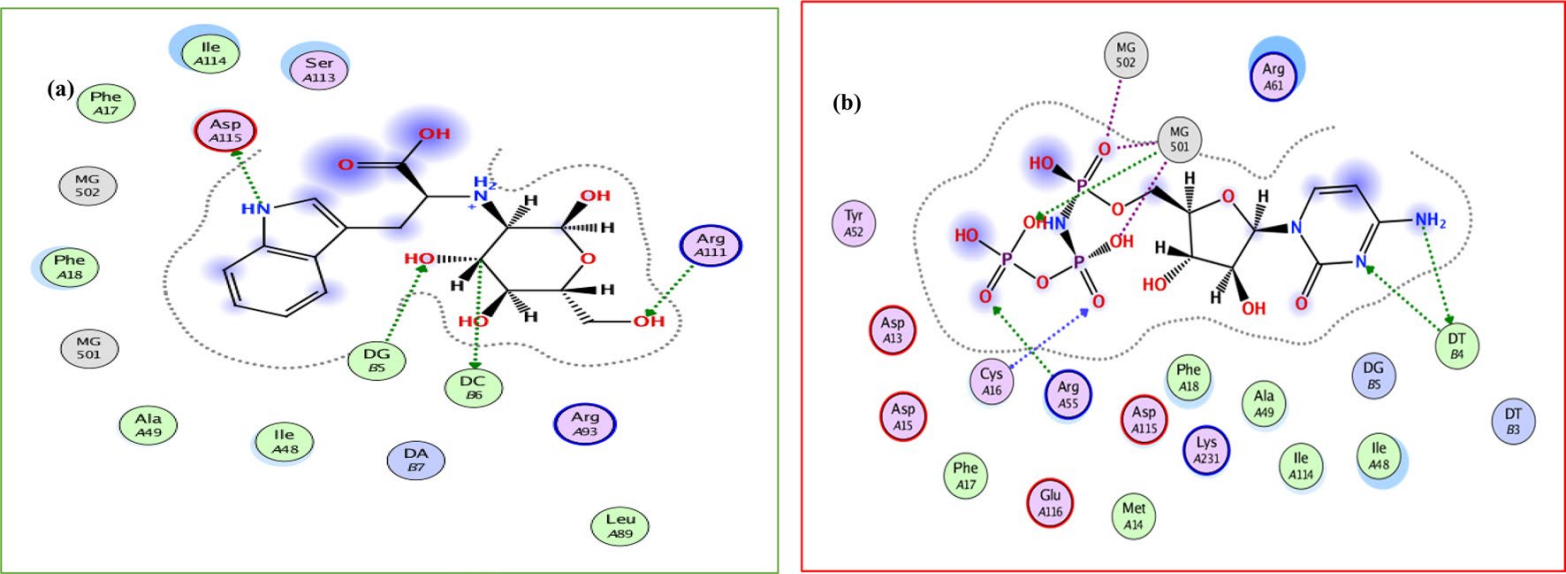
(**a**) The best pose of UNK4 in pocket site of receptors after 519 docking runs of Auto dock, (**b**) The best pose of CNP.

According to the results extracted from Tables 1 and 2, and Fig. 3, among our suggested structures, the structure most similar to CNP is UNK4 (see Table 1). We assumed the cyclic structure for D-fructose incorporated into UNK4, according to the results reported by Barclay et al.^54^. They reported that 99.5% of D-fructose in water is in cyclic form, and just 0.5% of its structure is in acyclic form. We also used Auto Dock 4.2.2 software^55^ to ensure that our grid boxes in auto dock/vina studies for UNK4 have been chosen correctly. The spacing between grid points was considered to be 0.375 Å, the grid box size was set at $126\times 126\times 126$ Å^3^, UNK4 as a ligand was considered to be flexible, and the grid searching was carried out at the local search genetic algorithm (LGA)^56,57^. 519 docking runs were performed to docking the ligand (UNK4) on the receptor (including the complex of DNA and polη). The results obtained in this part of our project were in good agreement with the Auto dock/vina results in that the target of UNK4 is the same as the target of CNP, as confirmed by interaction sites shown in Fig. 6. Figure 6a indicates that UNK4 is surrounded by the residues of DNA polymerase η (Polη), including phenylalanine 17, phenylalanine 18, Isoleucine 48, alanine 49, leucine 89, Arginine 93, arginine 111, serine 113, Isoleucine 114, and aspartic acid 115. Figure 6b shows that CNP is also surrounded by the same residues of DNA polymerase η (Polη), including aspartic acid 13, methionine 14, aspartic acid 15, Cysteine 16, phenylalanine 17, phenylalanine 18, alanine 49, Tyrosine 52, Arginine 55, Arginine 61, Isoleucine 114, aspartic acid 115, glutamic acid 116, and lysine 231, indicating that the active sites of UNK4 and CNP are very similar.

### Molecular dynamic simulation (MD)

MD simulations were carried out in four stages for two systems, including CNP and our designed structure (UNK4, see Table 1) complexed with their receptors (including DNA and DNA polymerase η). The topology files for UNK4, CNP, and the nucleic acids of DNA were constructed based on the Gromos 43 a1 force field using the PRODRG 2.x online server. The complexes were solvated by a layer of SPC water in all directions^58^. Our simulation box was considered a triclinic box with faces at least 1.0 nm apart from the closest atom of our system. The simulation box for each system involved at least 18,607 water molecules, 8 chloride ions, and 2 magnesium ions. MD simulation for minimization of each system was performed in three steps. The first step included the minimization of the whole system by applying the steepest descent. The second step included the conjugate gradient methods to minimize the system. The third minimization step was again the steepest descent method with emtol value of 100.0 kJ/mol.nm. Following minimization steps, the overall system was equilibrated in the NVT ensemble (where N, V, and T stand for the number of particles, volume, and temperature, respectively) for 500 ps (1 ps = 10^−12^ s) at 100 K. In this NVT ensemble, temperature-coupling times were 0.1 ps. The NPT ensemble followed this step (where N, P, and T stand for the number of particles, pressure, and temperature). The NPT equilibration was performed using md integrator for 1000 ps at 300 K and 1 bar. Berendsen algorithm was selected for both barostat and thermostat coupling algorithms in this step^58^. In the NPT ensemble, to increase the system's temperature from 100 to 300 K, the velocities were raised based on the Maxwell–Boltzmann distribution^59^. Finally, the MD production steps for each system was initiated for 70 ns (1 ns = 10^−9^ s) at 300 K with a time step of 1 fs (1 fs = 10^−15^ s) with 70 million steps in total. In this part, as we assumed that the overall 70 ns of MD simulation is enough to consider the various motions of protein including its folding as polymerase η is in complex with DNA and thus it is less flexible than free polymerase η. Parrinello–Rahman barostat^60^ and Nose–Hoover thermostat algorithms^60^ were used in MD simulation stages for pressure and temperature couplings, respectively. Furthermore, the periodic boundary condition was used for NPT simulation stage. To calculate van der Waals interactions, 'Cut-off' method was applied. In MD simulations, the van der Waals (rvdw) interactions were accompanied by neighbor list cut-off (rlist), where rvdw ≥ rlist. The frequency of updating the list of neighbors was considered 10 ps. In addition, the Particle Mesh Ewald (PME) model was exploited to estimate the electrostatic interactions^61,62^. For constraints on hydrogen bonds in systems, the LINCS algorithm was employed in all MD simulations^63^.

## Results and discussions

### Comparison of inhibitory effects of UNK4 and CNP

**Figure 7:**
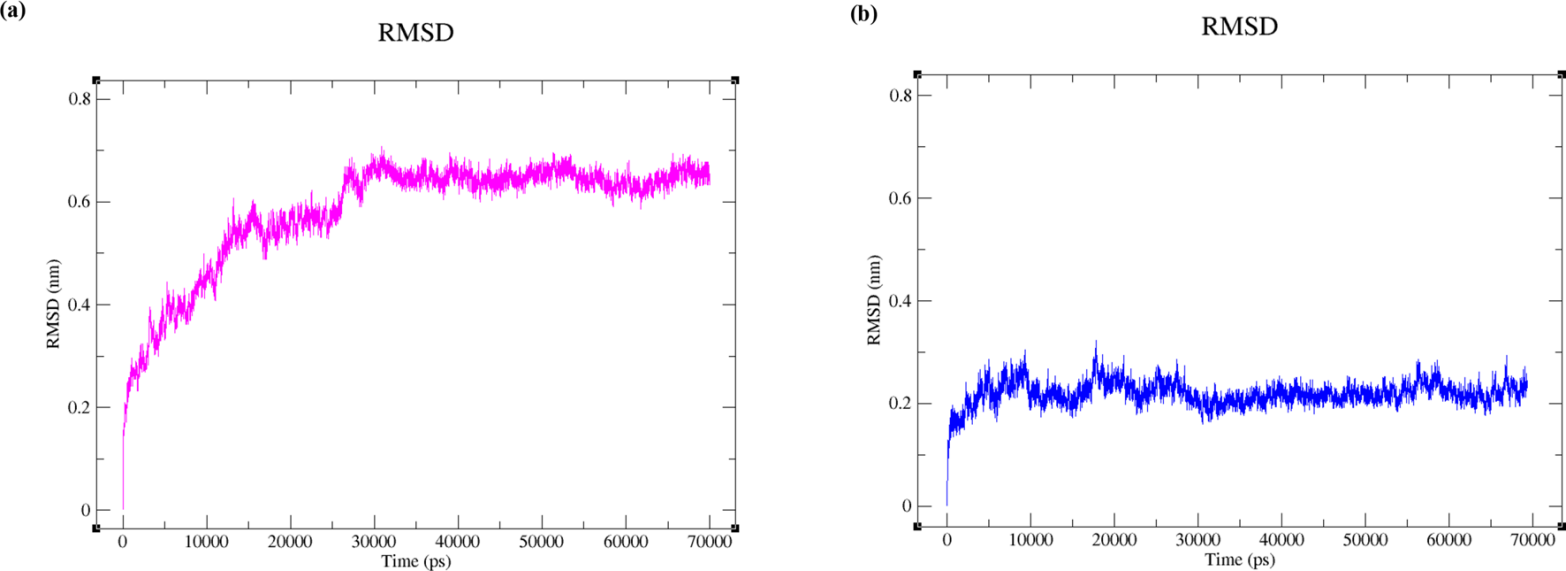
(**a**) Polymerase η backbone RMSD values in the presence of CNP (series 1, pink plot). (**b**) polymerase η backbone RMSD values in the presence of UNK4 (series 2, blue plot).

To explore the similarity between UNK4 and CNP in inhibition of their receptors (the complex of DNA and DNA polymerase η (Polη)), the patterns of the change in the root-mean-square deviation (RMSD) value of polymerase η during 70 ns of MD simulations were compared for these two designed (UNK4) and main (CNP) drugs. All the calculations of RMSD values of DNA and protein were performed with the aid of the GROMACS 5.2 package. Figure 7a,b indicate the patterns of RMSD values for the backbone of polymerase η in the presence of CNP and UNK4, respectively, during 70 ns of simulation. According to Fig. 7a, the significant changes in RMSD values of polymerase η can be observed in the first 15 ns of MD simulation. As can be seen in the Fig. 7a, the RMSD values for the backbone of polymerase η in the presence of CNP have been achieved at the 0.59 nm with respect to the reference structures (PDB ID: 6D0Z); however, after 20 ns of MD simulation, the backbone of polymerase η has fluctuated in the range of 0.6–0.7 nm. While in the system, including the complex of DNA and DNA polymerase η (Polη)) and UNK4 (Fig. 7b), in the primary 2.5 ns of MD simulations, the sharp changes in RMSD values with respect to the reference structures (PDB ID: 6D0Z) are observed. After the first 2.5 ns, the perseverance of RMSD values in MD simulation in Fig. 7b can be observed. RMSD plot in Fig. 7b indicates that the backbone of polymerase η has fluctuated in the range of 0.2–0.3 nm, indicating that the system, including the complex of DNA and Polη along with UNK4, was in an equilibrated and converged stage and kept stable between 2.5 and 70 ns of MD simulation.

**Figure 8:**
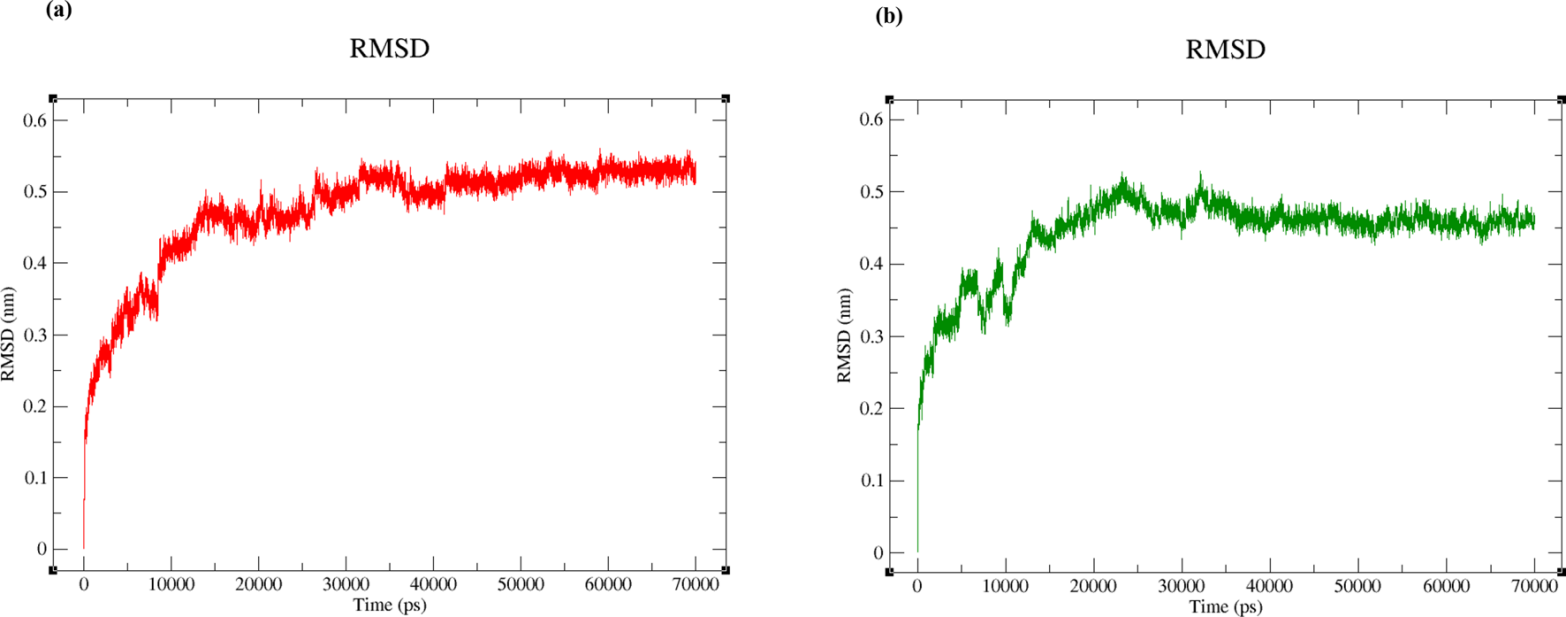
(**a**) RMSD values of DNA in the presence of CNP. (**b**) RMSD values of DNA in the presence of UNK4.

As DNA molecules were also considered to be part of the receptors, the same comparison was made between the changes in the root-mean-square deviation (RMSD) values of DNA in the presence of CNP (Fig. 8a) and UNK4 (Fig. 8b). As seen in Fig. 8a,b, the fluctuations in RMSD values of DNA with respect to the reference structures (PDB ID: 6D0Z) in both systems were sharp for the first 15 ns of MD simulations. The dynamic steadiness of DNA structure (for both systems) is observed between 15 and 70 ns. After the first 15 ns, both systems' perseverance of DNA RMSD values (Fig. 8a,b) are observed. RMSD plots for DNA also obviously indicate that the backbone of DNA has fluctuated in the range of 0.5–0.55 nm in the complex of DNA and Polη along with CNP (Fig. 8a). The backbone of DNA has fluctuated in the range of 0.45–0.5 nm in the complex of DNA and Polη along with UNK4 (Fig. 8b), suggesting that both systems were in an equilibrated and converged stage and kept stable between 15 and 70 ns of MD simulations.

**Figure 9:**
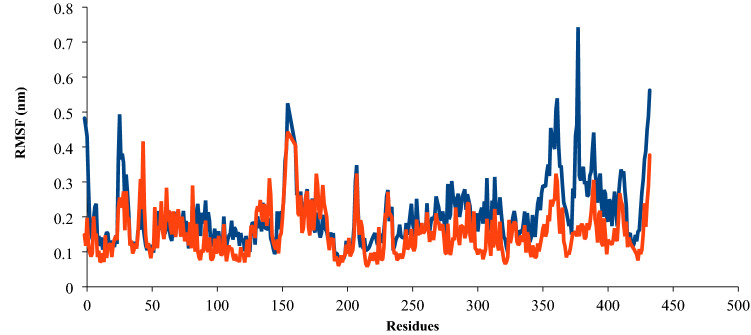
RMSF plots of the protein backbone Polη complexed with CNP (series 1, blue plot) and UNK4 (series 2, red plot) during 70 ns MD simulation.

RMSF (root mean square fluctuation) of protein versus its residue was plotted for both systems to characterize further the dynamic behaviour or structural flexibility of the above-described systems (Fig. 9). The blue and red plots in Fig. 9 correspond to the Polymerase η complexed with CNP and UNK4, respectively. Polymerase η (Polη) has the template-primer, including palm (including the residues 1–13 and 90–238), fingers (including the residues 17–87), and thumb (including the residues 289–378) as well as the little finger domain of polymerase η or polymerase associated domain (PAD: the residues 319–432), which especially belongs to Y-family polymerases^9^. As discussed above, in Fig. 6a,b, the main residues of Polη interacting with UNK4 and CNP are phenylalanine18, Isoleucine 48, and alanine 49, Isoleucine114, and aspartic acid 115. These residues mainly belong to the palm and finger domains of Polη. As seen in Fig. 9, in series 1, RMSF values of the residues 1–50 in the presence of CNP vary in the range of 0.116–0.49 nm. While in series 2, RMSF values of the residues 1–50 in the presence of UNK4 vary in the range of 0.085–0.41 nm. This analysis shows that the fingers and palm domains of polymerase η in CNP and UNK4 have similar fluctuations over 70 ns of MD simulation.

**Figure 10:**
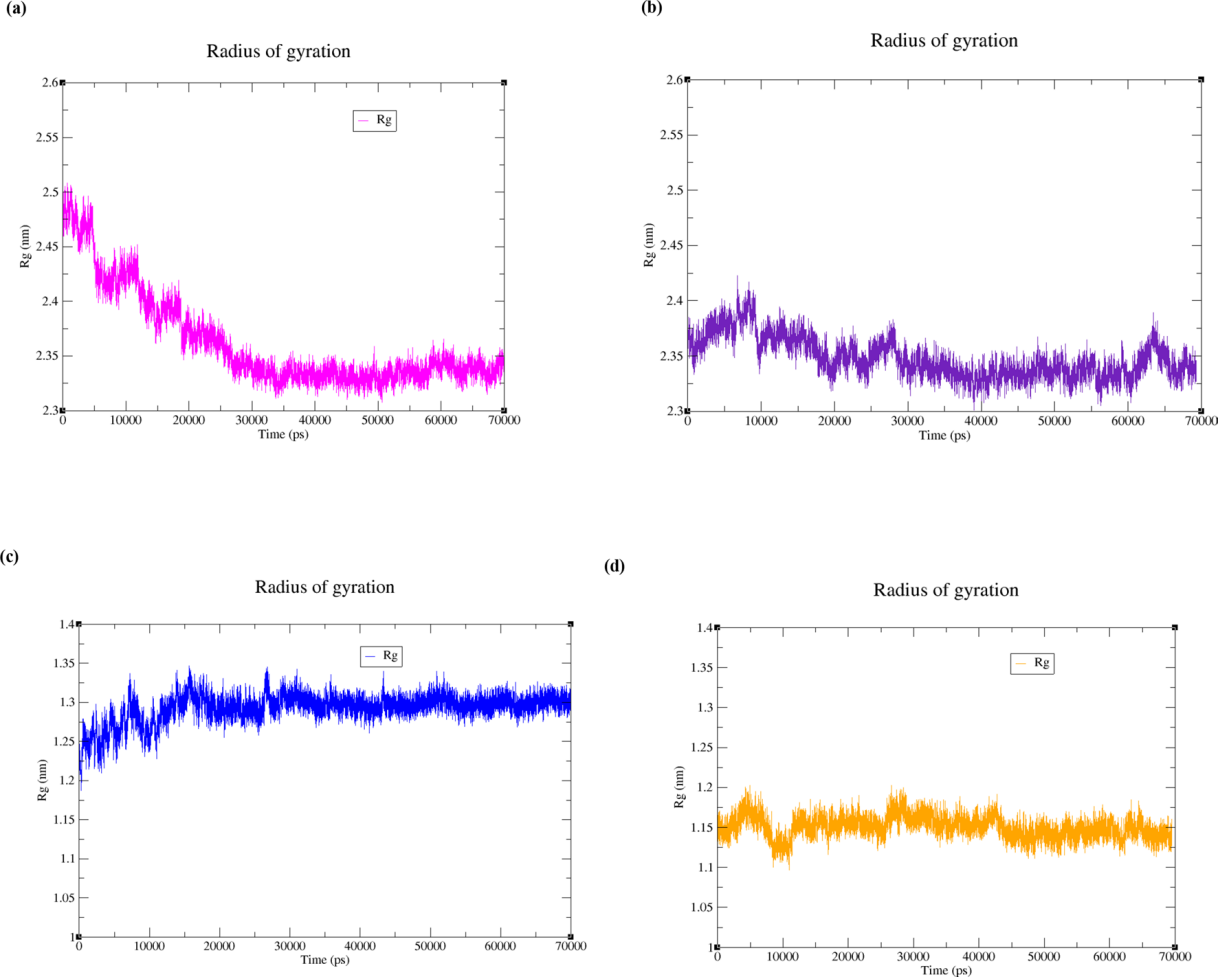
(**a**) Rg plot of protein in the presence of CNP. (**b**) Rg plot of protein in the presence of UNK4. (**c**) Rg plot of DNA strands in the presence of CNP. (**d**) Rg plot of of DNA strands in the presence of UNK4.

To evaluate the compactness of systems in MD simulations, we considered the changes in the radius of gyration (Rg) for the protein Polη complexed with CNP (Fig. 10a) and UNK4 (Fig. 10b). According to Fig. 10a, the radius of gyrations of polymerase η in the presence of CNP is in the range of 2.35–2.50 nm. Figure 10b depicts that the radius of gyrations of polymerase η in the presence of UNK4 is in the range of 2.3–2.40 nm. Figure 10c,d represent the change of radius of gyration of DNA in the presence of CNP and UNK4, which is in the range of 1.25–1.325 nm for the system CNP (Fig. 10c) and is in the range of 1.1–1.2 in the system UNK4, (Fig. 10d).

**Figure 11:**
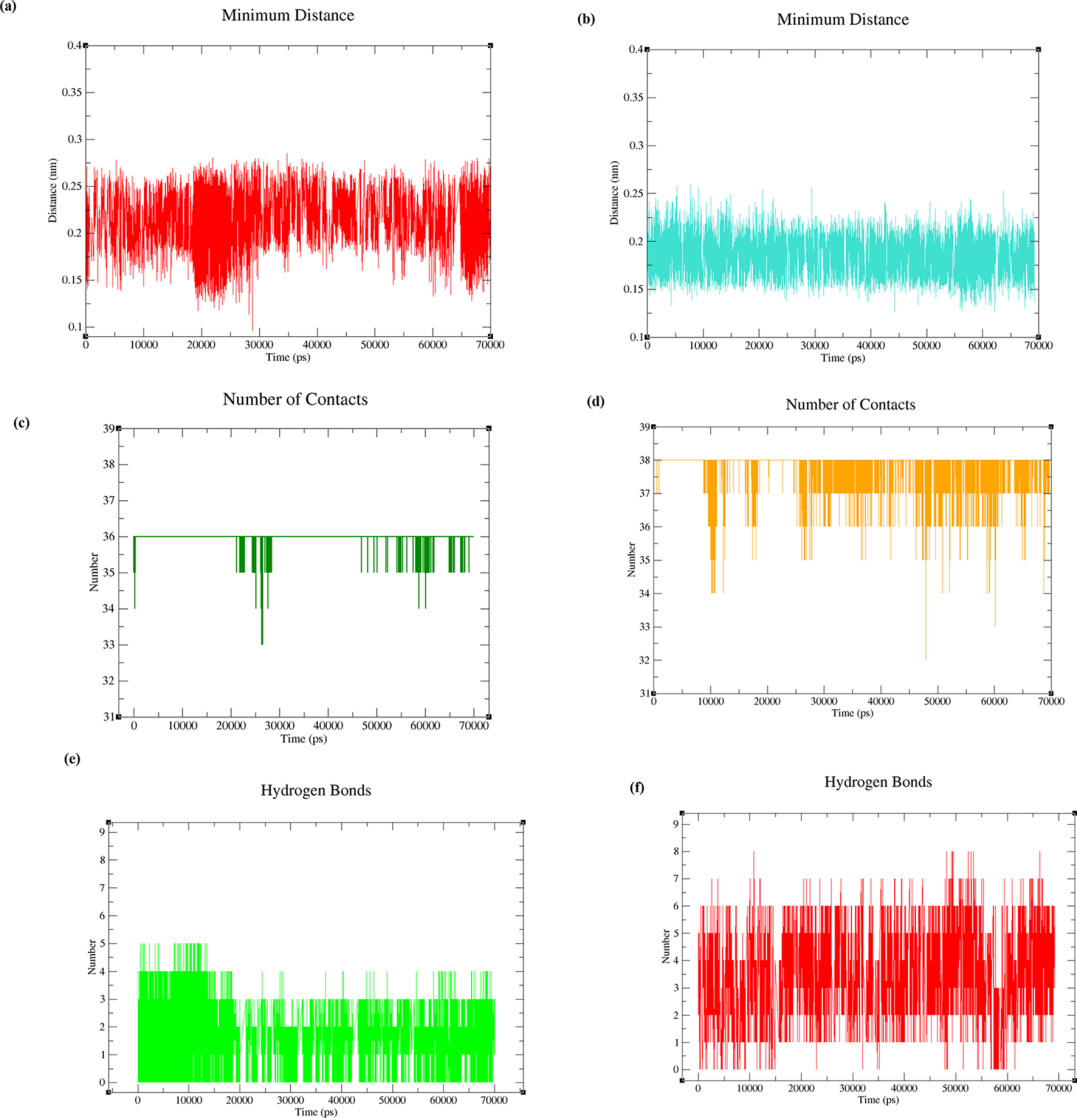
Plots of the protein backbone (polη) complexed with CNP and UNK4 during 70 ns MD simulation: (**a**) plot of average of the minimum distance between polη and CNP, (**b**) plot of average of the minimum distance between polη and UNK4, (**c**) plot of number of contacts between polη and CNP, (**d**) plot of number of contacts between polη and UNK4. The intermolecular hydrogen-bonding distribution plot versus time for (**e**) polη–CNP and (**f**) polη-UNK4.

As seen in Fig. 11a,b, the minimum distances between polymerase η and CNP (Fig. 11a) and UNK4 (Fig. 11b) during 70 ns of MD simulations are acceptable in both systems, as they are < 0.25 nm in most of the frames. Besides, under 6 Å, the number of contacts between polymerase η and UNK4 (Fig. 11d) is more than that between polymerase η and CNP (Fig. 11c). The intermolecular hydrogen bond analyses (Fig. 11e,f) display a possibility of forming a maximum of 7 and 8 hydrogen bonds between polymerase η and UNK4 (Fig. 11f). In contrast, the maximum number of hydrogen bonds between polymerase η and CNP is estimated to be 5 and 4 (Fig. 11e).

**Figure 12:**
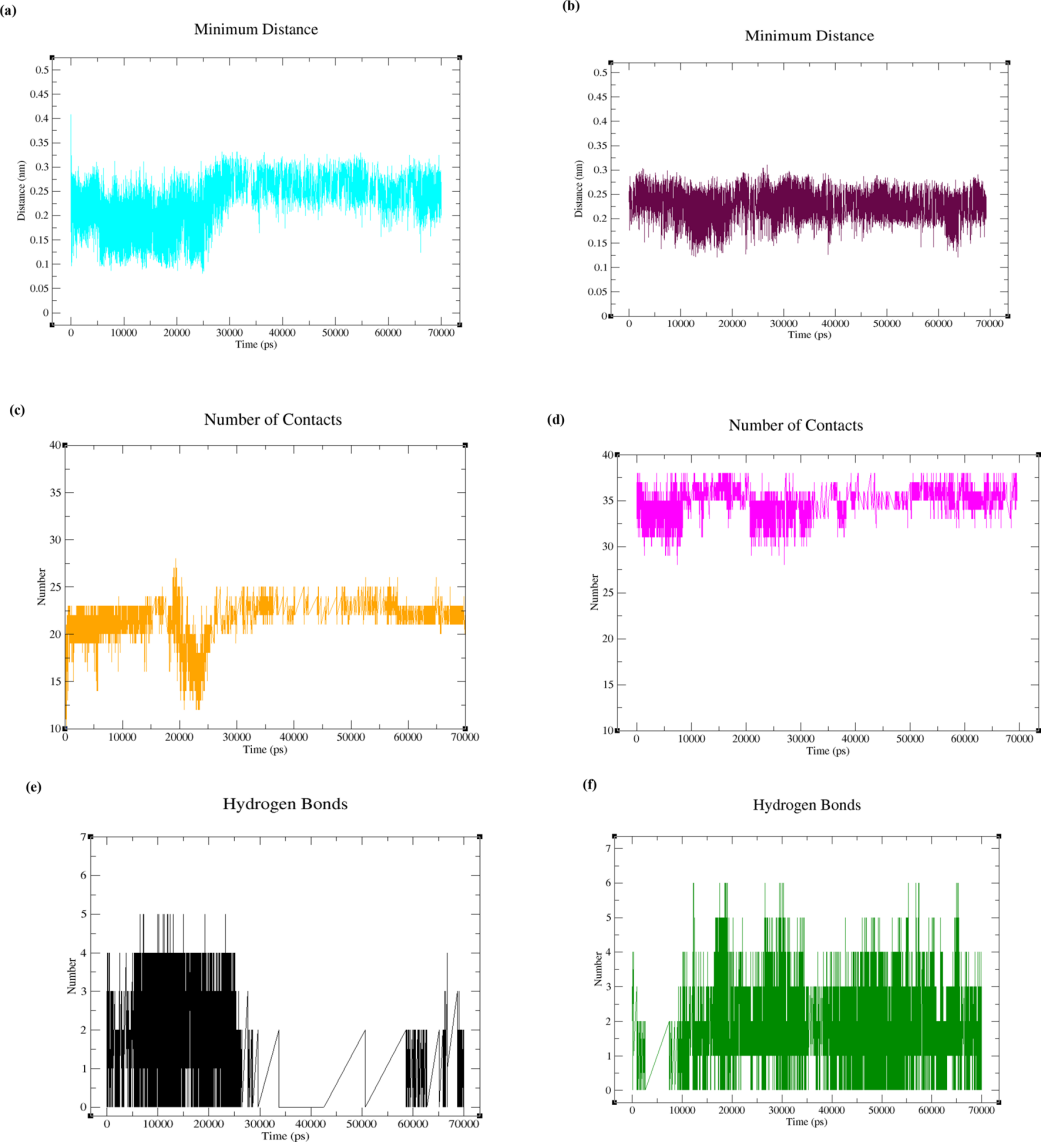
Plots of the DNA backbone complexed with CNP and UNK4 complexes during 70 ns MD simulation: (**a**) plot of average of the minimum distance between DNA and CNP, (**b**) plot of average of the minimum distance between DNA and UNK4, (**c**) plot of number of contacts between DNA and CNP, (**d**) plot of number of contacts between DNA and UNK4. The intermolecular hydrogen-bonding distribution plot versus time for (**e**) DNA–CNP and (**f**) DNA-UNK4.

As seen in Fig. 12a,b, the minimum distances between DNA and CNP (Fig. 12a) as well as between DNA and UNK4 (Fig. 12b) during 70 ns of MD simulations are < 0.25 nm in most of the frames. Figure 12c,d show that the number of contacts between DNA and UNK4 is more than that between DNA and CNP. The intermolecular hydrogen bond analyses (Fig. 12e,f) show a maximum of 5 hydrogen bonds between DNA and CNP (Fig. 12e) and a maximum number of 6 hydrogen bonds between DNA and UNK4 (Fig. 12f).

To ensure that the structure of UNK4 was reliable during 70 ns of molecular dynamics simulation, the RMSD of UNK4 with respect to the optimized geometry of the lowest energy conformer of UNK4 at B3LYP/6‐311++G (d, p) level was plotted against time (ps). As seen in Fig. S4, the RMSD plots for UNK4 indicates that the backbone of this drug has fluctuated in the range of 0–0.1 nm, which illustrates that this ligand has been correctly parametrized for MD simulation. In addition, eight snapshots during the first 30 ns of MD simulation were taken for both CNP (Fig. S5) and UNK4 (Fig. S6) complexed with Polη and DNA. According to Figs. S5 and S6, UNK4, compared to CNP, can have a more prominent effect on the complex of Polη and DNA. In these Figs. S5 and S6, Mg ions are shown in pink color, and chloride ions are yellow. Two strands are shown in cyan and blue colors. H-bond model in red displays solvent (water molecules), Polη is shown using the new cartoon style, and both UNK4 and CNP are shown by the ball and stick model.

### MM/PBSA calculation of UNK4 and CNP on receptors (DNA and polymerase η)

The binding free energies of UNK4 and CNP on the receptor (DNA and polymerase η) were obtained by considering the Molecular Mechanics/Poisson–Boltzmann Surface Area (MM/PBSA) method during a 70 ns period of MD trajectory (for these calculations, 179 snapshots in both mentioned systems were chosen). The energy terms calculated by the g_mmpbsa package are listed in Table 3^64^. MM/PBSA results illustrate that the binding energy of UNK4 to DNA (− 70.14 kJ/mol) is more negative than that of CNP to DNA (− 55.81 kJ/mol). Additionally, according to our calculations, UNK4 can bind to the polymerase η with the binding energy of − 144.99 kJ/mol, indicating the stronger binding of UNK4 with polymerase η, as compared with the binding energy of CNP with polymerase η (− 125.16 kJ/mol). According to our results, the binding energy values of both CNP and UNK4 are negative, and their interactions with the receptor (DNA and polymerase η) are thermodynamically very favourable. As a result, UNK4, with the volume, area, and electron density similar to CNP, could be considered an alternative anticancer drug for Cytarabine with much fewer side effects because its building blocks are amino acid and cyclic carbohydrate.

**Table 3:** Various energy terms obtained through MM/PBSA calculations.

MM/PBSA	DNA and CNP	polymerase η and CNP	DNA and UNK4	polymerase η and UNK4
van der Waals energy (kJ/mol)	− 67.02	− 217.02	− 80.75	− 158.17
Electrostatic energy(kJ/mol)	− 2.15	− 11.32	− 6.57	− 146.62
Polar solvation energy(kJ/mol)	19.25	117.84	24.57	175.83
Non-polar solvation energy (kJ/mol)	− 5.89	− 14.66	− 7.39	− 16.03
Binding energy (kJ/mol)	− 55.81 ± 17.58	− 125.16 ± 35.68	− 70.14 ± 10.74	− 144.99 ± 18.94

### Evaluations of the validity of our theoretical calculations with respects to the experimental data

To validate our theoretical studies on UNK4 as an anticancer drug, we studied the interaction of UNK4 with SI/II-pocket of KRAS protein using Auto Dock/Vina. The experimental data of the complexes of this protein with different drugs protein are reported with the PDB ID codes, including 6GJ5, 6GJ6, 6GJ7, and 6GJ8. In Table 4, entries 1–4 indicate the structures of inhibitors (drugs) of the SI/II-pocket of KRAS protein extracted from their PDB files. Dirk Kessler et al. first introduced these inhibitors^65^, The SI/II-pocket of KRAS protein was selected for three reasons. First, KRAS protein plays an essential role in activating various signaling molecules that permit the transduction of signals from the cell surface to the nucleus. In turn, these processes can control different stages of the cell cycle, such as chemotaxis^66^, growth, and apoptosis^67,68^. Secondly, KRAS with the mutation is known as a prominent driver of human cancers^69^. Thirdly, the inhibitors indicated in entries 1–4 in Table 4 contain indole substructures, and they have been reported to have nanomolar binding affinity to the SI/II-pocket of KRAS protein^65^. As UNK4 has indole fragment in its structure, we examined UNK4 inhibitory activities against KRAS to support our theoretical results.

**Table 4:**
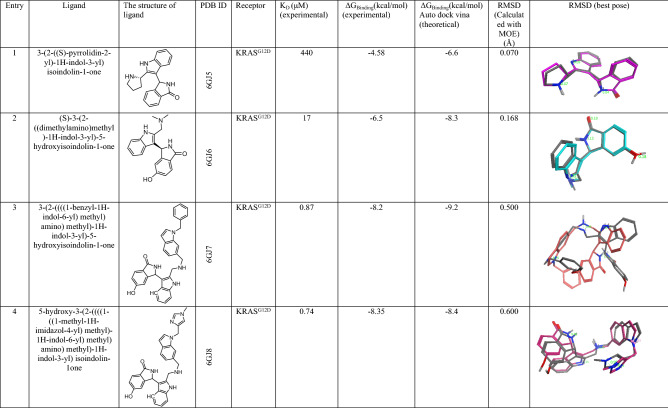
The calculated and experimental ΔG_Binding_ (kcal/mol) values of some known compounds toward KRAS protein.

The experimental binding affinities of KRAS toward these known inhibitors were estimated from measured dissociation constants K_D_^70^, and by employing Eqs. (3) and (4).

3 $$ \Delta {\text{G}}_{{{\text{Dissociation}}}} = - {\text{RTLn K}}_{{\text{D}}} $$

4 $$ \Delta {\text{G}}_{{{\text{Dissociation}}}} = - \Delta {\text{G}}_{{{\text{binding}}}} $$

**Figure 13:**
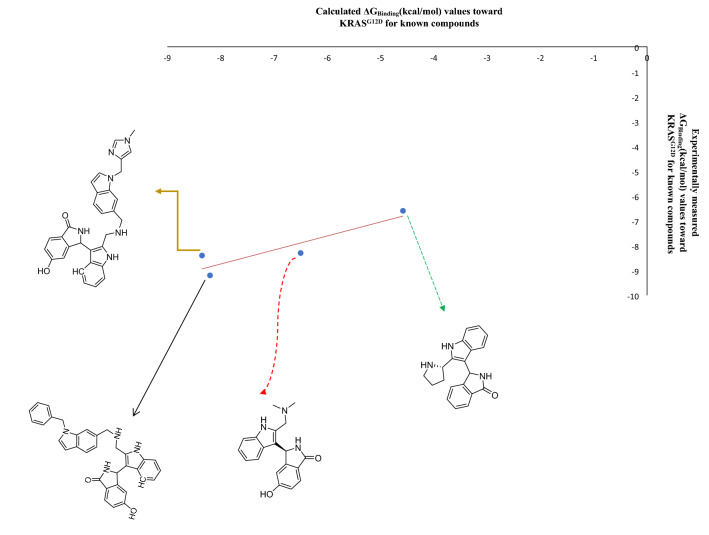
Plot of the experimental ΔG_Binding_ (kcal/mol) values against the calculated ΔG_Binding_ (kcal/mol) values for the known KRAS inhibitors.

In this part, we calculated the binding affinity of the SI/II-pocket of KRAS protein toward these inhibitors (entries 1–4 in Table 4) and UNK4 using the Auto Dock/Vina plugin with scripts from the Auto Dock Tools Package. We then obtained the linear graph shown in Fig. 13 by plotting these calculated binding affinities versus their experimentally binding affinities (see Table 4 for calculated and experimental binding affinity values). Using the linear equation obtained from this linear graph ($y = 0.564x - 4.23,\; R^{2 } = 0.828$) and the binding affinity of KRAS for UNK4 obtained by Auto Dock/Vina, we predicted the experimental binding affinity of KRAS for UNK4 to be − 8.1 kcal/mol, which is in good agreement with the binding affinity of KRAS for UNK4 calculated by Auto Dock/Vina (− 6.9 kcal/mol).

## Synthesis of UNK4

In this section, we used the approach reported by Chanda et al.^32^ for the synthesis of UNK4 (Table 1, entry 6). In a round bottom flask containing 7 mL MeOH and 0.5 mL of acetic acid, tryptophan (1 mmol) was added, and D-fructose (1 mmol) was loaded into this flask. Proceeding this stage, Zn (OAc)_2_ (1 mg) was added to the reaction environment followed by the reflux of the reaction mixture between 6 and 12 h. During the progress of the mentioned reaction, the mixture turned reddish-brown colour. In the last steps the received solution was filtered through Sintered glass filter disc under the influence of a partial vacuum. After being concentrated with the aid of a rotary evaporator, the filtrate was washed with acetone four times (20 mL in total), and the obtained crystal was again decanted with water and distilled ethyl acetate. Then, the organic phase was separated from the aqueous phase and subjected to vacuum. The recrystallized product was dried under vacuum. The FT-IR spectrum and H-NMR data of the product UNK4 are given in Fig. S7 and Table S2. In the next section, we report the measured log P of the synthesized UNK4.

## Experimental measurement of log P of UNK4

**Figure 14:**
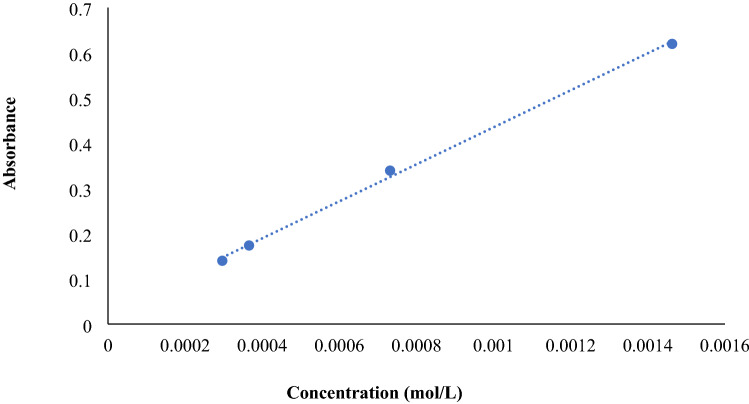
The UV calibration curve of UNK4.

To obtain log P of UNK4, this compound was dissolved in water to make the solutions with known concentrations, including 0.0002964 M, 0.0003658 M, 0.0007316 M and 0.0014632 M. Subsequently, the absorptions of the mentioned solutions were scanned between 200 and 800 nm. Then, the calibration curve for UNK4 was plotted at the λ_max_ 280 nm. Figure 14 illustrates the UV calibration curve with $y=409.4x+0.0258 , {R}^{2 }=0.9981$. The y-axis in Fig. 14 displays the absorbance of each solution, and the x-axis depicts their concentrations. The UV spectra of the solutions mentioned above can be found in Fig. S8.

With a calibration curve in hand, 10 mL of the UNK4 solutions with the concentrations of 0.0014632 M, 0.0002964 M and 0.0007316 were added to 3 separatory funnels. Then, 10 mL of 1-Octanol was loaded into each of the funnels and the funnels were shaken. Then, mixture in each funnel was allowed to achieve equilibrium in 2 phases. Then absorbance of each aqueous solution was again obtained at the λ_max_ 280 nm to measure concentration of UNK4 in water using the calibration curve described above (Fig. 14 and Table S3). Using the Eqs. (5)–(8)^71^, we measured the experimental value of log P for UNK4. In Eq. (5), C_aq, Init_ represents the initial concentration of the aqueous solution of UNK4, while C_aq, eq_ and C_Oct,eq_ show the concentrations of UNK4 in the aqueous and octanol solutions after equilibration, respectively; V_oct_ and V_aq_ are the epithets of the volumes of aqueous solution and 1-Octanol in the separatory funnels. According to the mass balance, the relationship between C_aq, Init_, C_aq, eq_ and C_Oct, eq_ can be explained by Eq. (5). The Eq. (6) can result from the Eq. (5) and *K*_*ow*_^72^ represents the ratio of the concentrations of a compound between 1-Octanol and water, which can be expressed as Eq. (7). Using the Eqs. (7) and (8), log P of the UNK4 solutions were measured to be − 0.78, − 1.61 and − 1.52 with the average value of − 1.3. A good correlation exists between this experimentally measured log P (-1.3) and theoretically calculated log P of UNK4 (− 1.64) obtained in Section “Prediction of log P for the designed anticancer structure”.

5 $$ C_{aq, Init} V_{aq } = C_{aq, eq} V_{aq } + C_{Oct, eq} V_{Oct} $$

$$ V_{aq } = V_{Oct} $$

6 $$ C_{aq, Init} - C_{aq, eq} = C_{Oct, eq} $$

7 $$ K_{OW} = \frac{{C_{Oct, eq} }}{{C_{aq, eq} }} = \frac{{C_{aq, Init} - C_{aq, eq} }}{{C_{aq, eq} }} $$

8 $$ \log K_{OW} = \log P $$

The comparison of our experimentally measured log P of UNK4 (− 1.3) with the experimentally reported log P of Cytarabine (− 2.8)^51^ indicate that the suggested structure of UNK4 is comparable to the main drug (Cytarabine) in terms of cell membrane permeability.

## Conclusion

According to many studies, carbohydrates are involved in many cells’ recognition processes^73–75^. These carbohydrate structures bear high polarities that can affect their pharmacokinetic properties as a drug. In this study, we designed the new structures containing both carbohydrates and amino acids and explored their interactions with the combined receptor containing the DNA and polymerase η (polη). We chose small molecules composed of D-fructose and amino acids such as valine, arginine, isoleucine, leucine, tryptophan, alanine, and phenylalanine. These small designed molecules should have acceptable solvation in both lipophilic and hydrophilic media. Therefore, they might achieve their targets in the human cells quickly. These newly designed amino acid-carbohydrate-based structures should have fewer toxic effects in high dosage as new anticancer drugs. We used in silico studies to find the best candidates for these newly designed drugs. Our results indicated that designed UNK4 structure (Table 1) could be a suitable replacement for Cytarabine or other traditional drugs used to inhibit polη, which plays an essential role in the replication of damaged DNA in the cancerous cell and leads to the formation of daughter cells having the genome with some lesions. This process will be repeated over and over and result in the accumulation of mutations and tumorigenesis. To stop this replication process in the tumor cells, in this study, both DNA and polη were considered the combined receptor. As Cytarabine is one of the well-known drugs to stop the replication process of DNA in tumor cells, the first goal was to design a structure most similar to Cytarabine, but with fewer side effects. Thus, the physical properties such as volumes, areas, and the electrostatic potential maps of the newly designed amino acid-carbohydrate-based structures were obtained and compared with those of Cytarabine. We selected the candidate structures which were very similar to Cytarabine in terms of these physical properties. Then, the docking procedures were performed to predict the bound conformations and the binding affinities of Cytarabine and UNK4 with the combined receptor of DNA and polη using the Auto Dock/Vina plugin with scripts from the Auto Dock Tools Package. It was found that UNK4 has an excellent binding affinity towards the receptor of DNA and polη (− 9.2 kcal/mol), in comparison with the binding affinity of CNP toward this receptor (− 8.8 kcal/mol). DFT calculations in combination with a solvation model based on density (SMD) (refer to Table 2)^49^ were employed to calculate log P values of Cytarabine and UNK4. The log P for UNK4 was found to be − 1.64 comparable to the reported experimental log P value of Cytarabine (− 2.8). Finally, MD simulations were carried out for the CNP and UNK4 complexed with their receptor (DNA and Polη) to comprehend their inhibition capability better to replicate DNA. MD simulations and its energy analysis performed by MM/PBSA calculations indicated that UNK4, containing the natural building blocks of amino acid and carbohydrate, could be a promising inhibitor for the combined receptor of DNA and polη, and thus a suitable replacement for Cytarabine.

## Supplementary Information

Supplementary Information 1.

Supplementary Information 2.

Supplementary Information 3.

Supplementary Information 4.

Supplementary Information 5.

Supplementary Information 6.

## Data Availability

All data generated or analysed during this study are included in this published article [and its supplementary information files].
