# Supplementary material for: Design of amino acid- and carbohydrate-based anticancer drugs to inhibit polymerase η
Source: Sci Rep. 2022 Nov 2;12:18461. doi: 10.1038/s41598-022-22810-z (PMC9630280; doi:10.1038/s41598-022-22810-z)
Supplement: Supplementary file 1 — Supplementary Information 1. [file 41598_2022_22810_MOESM1_ESM.pdf]

Design of Amino Acid- and Carbohydrate-Based Anticancer Drugs to inhibit polymerase η

Sepideh Kalhor, Alireza Fattahi*

Department of Chemistry, Sharif University of Technology, Tehran, Iran

* Corresponding Author: fattahi@sharif.edu

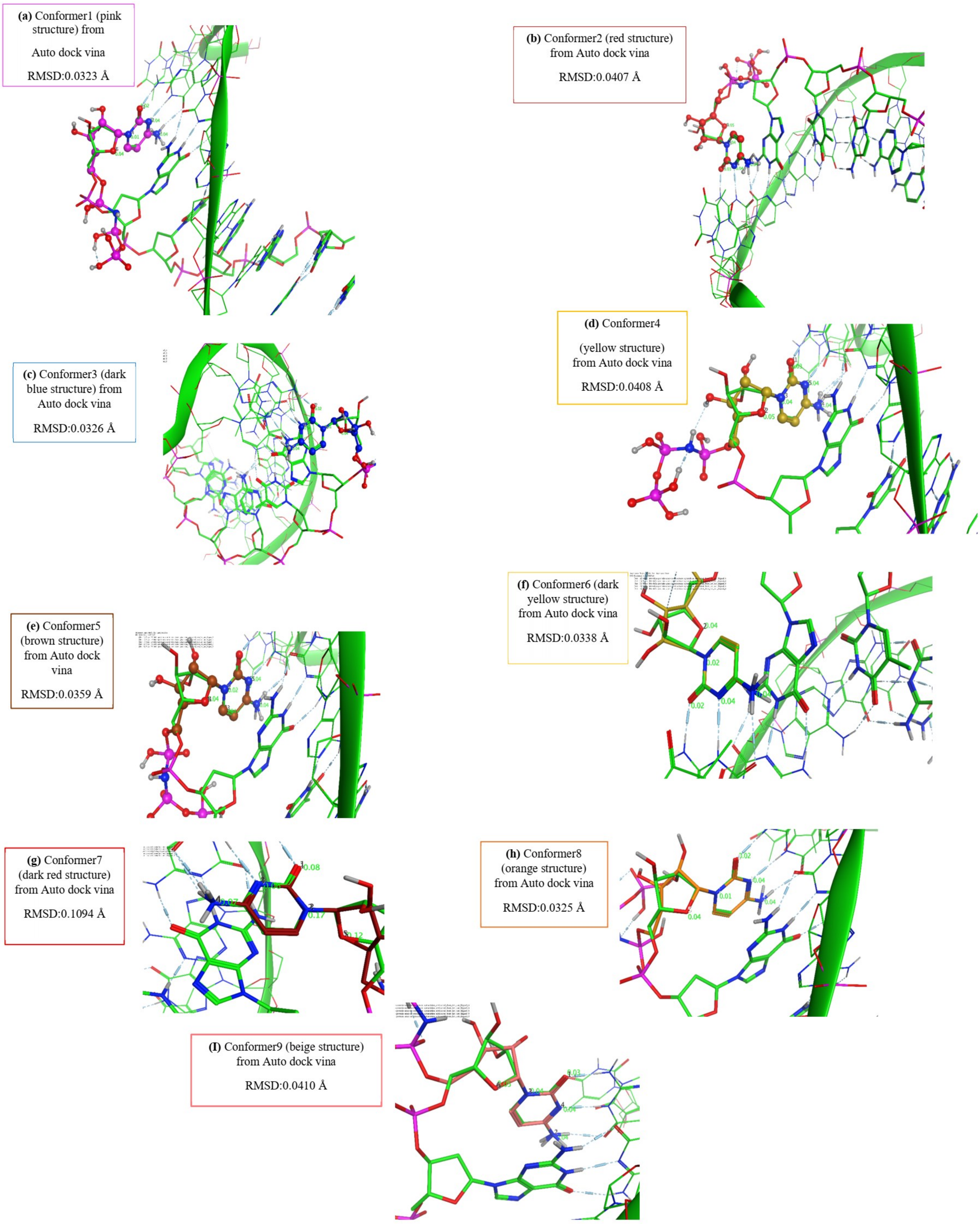

Figure S1. The structural information (obtained by Auto Dock Vina) for all 9 conformers of CNP complexed With DNA and DNA polymerase η (Polη) with the least RMSD = 0.0323 Å (structure of Polη not shown here) (MOE 2018).

Table S1. Possible off-target interactions of UNK4 according to Auto Dock Vina studies

Entry	Name of ligand	Structure of ligand	PDB ID	Receptor	AFFINTY (kcal/mol)
**1**	cytarabine	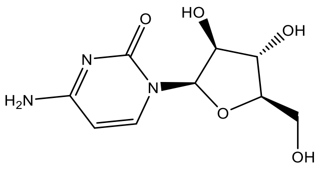	6D0Z	complex of protein and DNA	-6.6
**2**	cytarabine	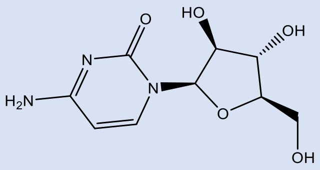	3mdc	Polymerase λ	-7.9
**3**	UNK4	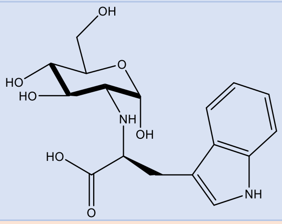	3mdc	Polymerase λ	-8.5
**4**	cytarabine	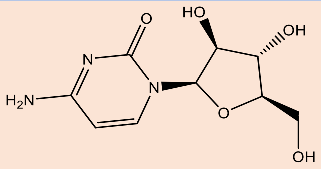	1P5Z	Human deoxyribonucleoside kinase	-7.2
**5**	UNK4	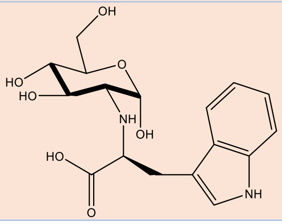	1P5Z	Human deoxyribonucleoside kinase	-7.7

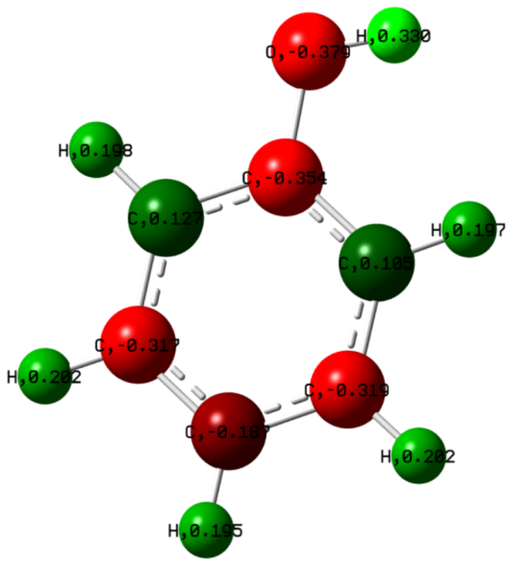

(a) The opted geometry of **Phenol** in water solvent

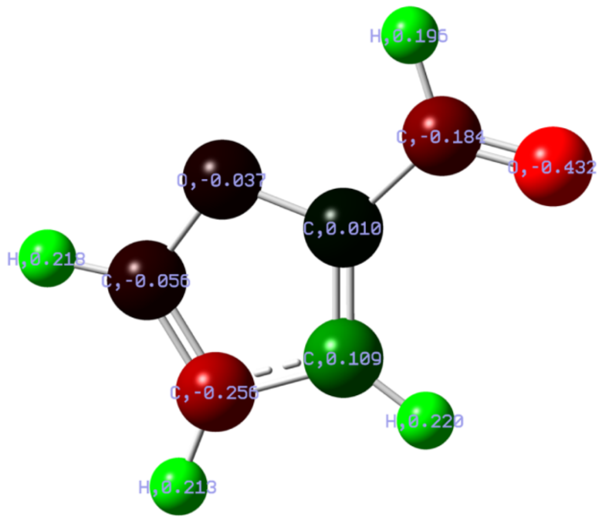

(b) The opted geometry of **Furfural** in water solvent

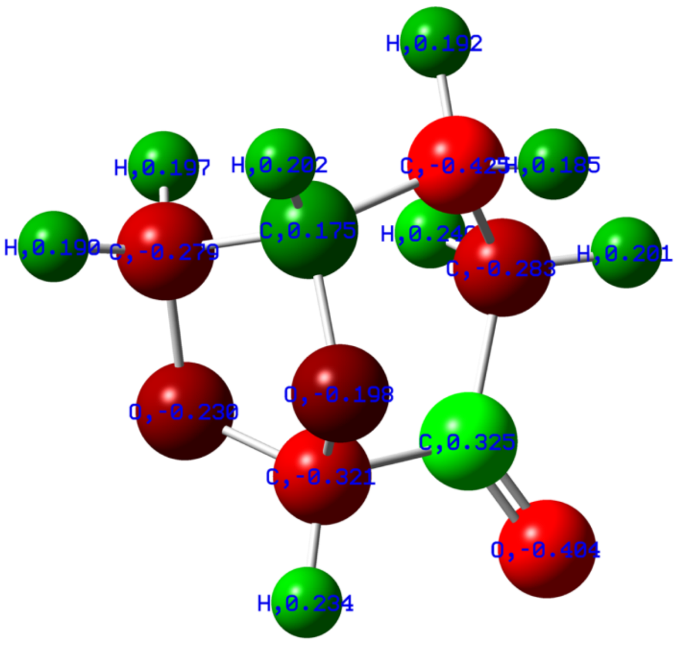

(c) The opted geometry of **Dihydrolevoglucosenone** in water solvent

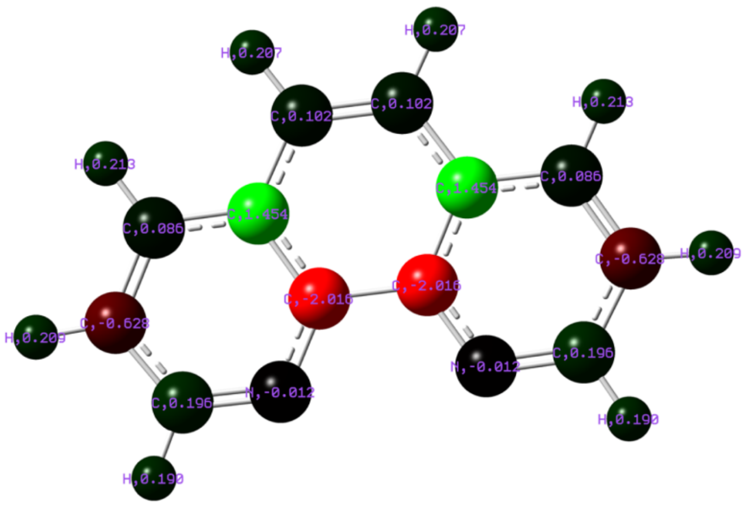

(d) The opted geometry of **Phenanthroline** in water solvent

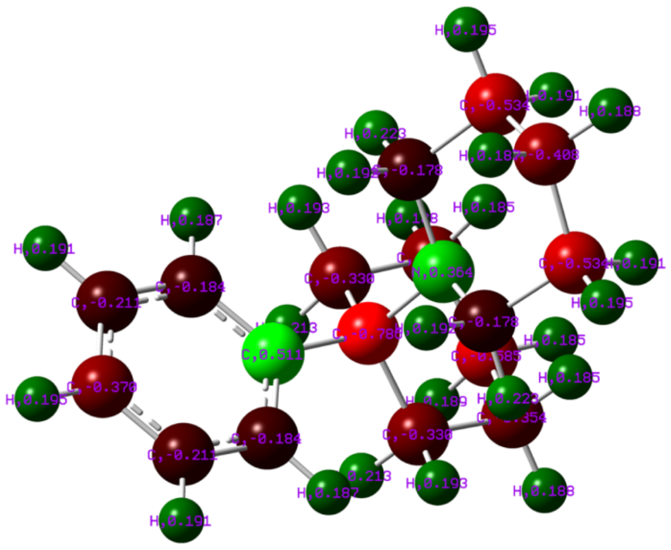

(e) The opted geometry of **Phencyclidine** in water solvent

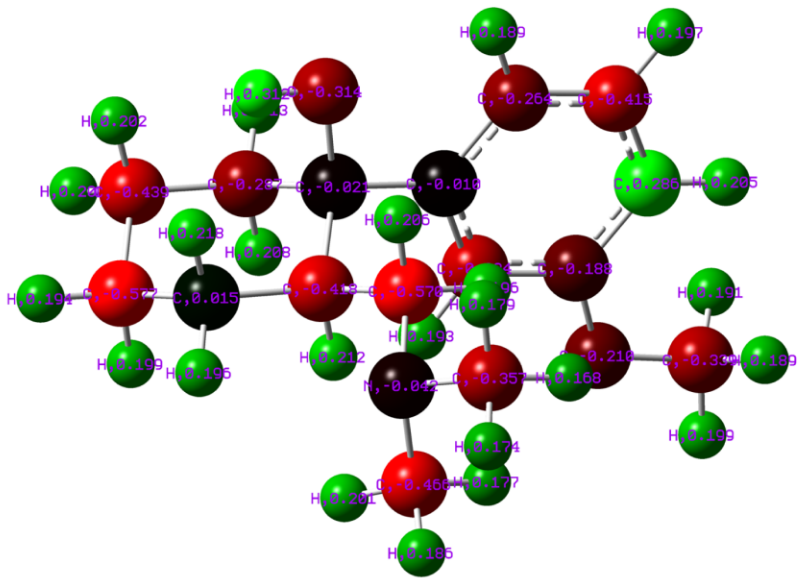

(f) The opted geometry of **Tramadol** in water solvent

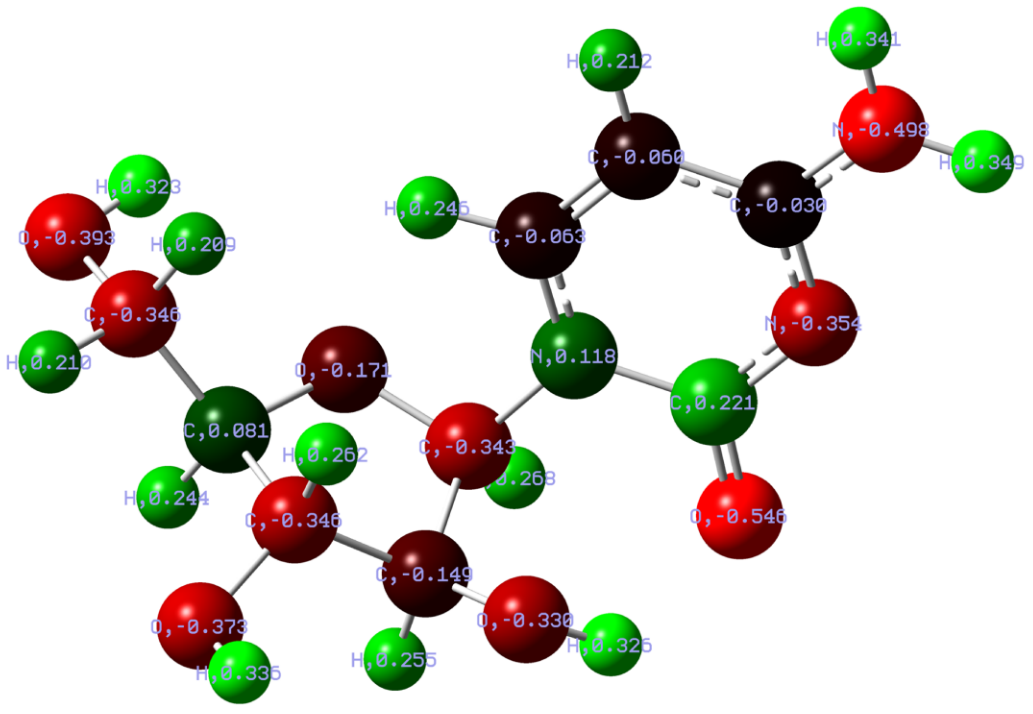

(g) The opted geometry of **Cytarabine** in water solvent

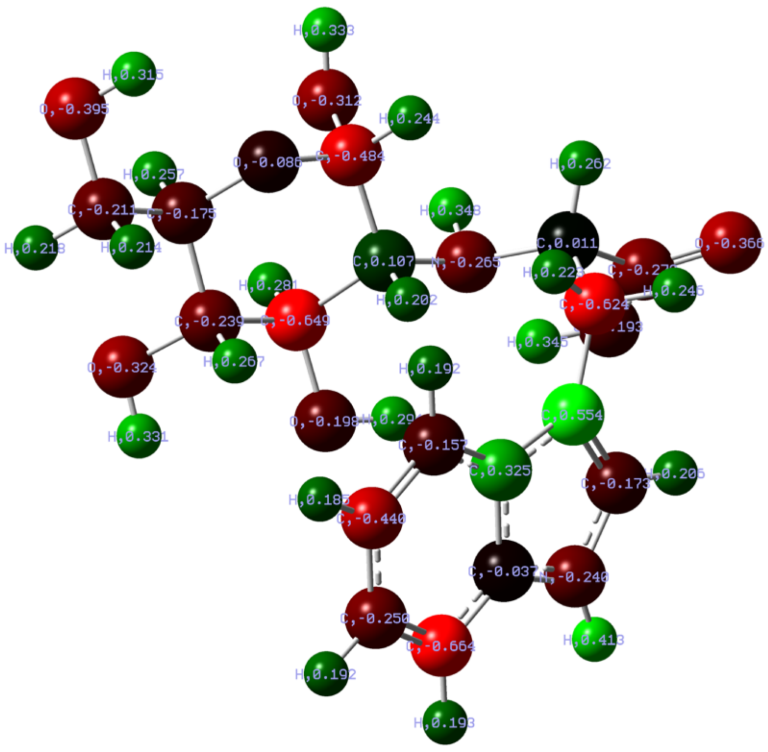

(h) The opted geometry of **UNK4** in water solvent

Figure S2. Optimized geometries of the lowest energy conformers of the known compounds including **(a)** Phenol, **(b)** Furfural, **(c)** Dihydrolevoglucosenone, and some known drugs such as **(d)** Phenanthroline, **(e)** Phencyclidine, **(f)** Tramadol, **(g)** Cytarabine and **(h)** UNK4 at the M06-2X/6-311+G (d, p) level in water solvent (gauss view6).
