# Supplementary material for: Design of amino acid- and carbohydrate-based anticancer drugs to inhibit polymerase η
Source: Sci Rep. 2022 Nov 2;12:18461. doi: 10.1038/s41598-022-22810-z (PMC9630280; doi:10.1038/s41598-022-22810-z)
Supplement: Supplementary file 2 — Supplementary Information 2. [file 41598_2022_22810_MOESM2_ESM.pdf]

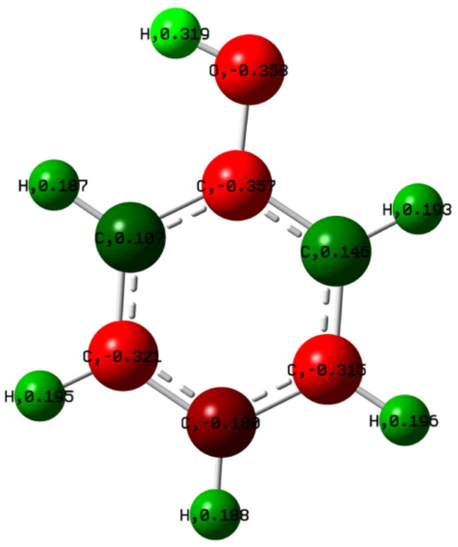

(a) The opted geometry of Phenol in 1-Octanol solvent

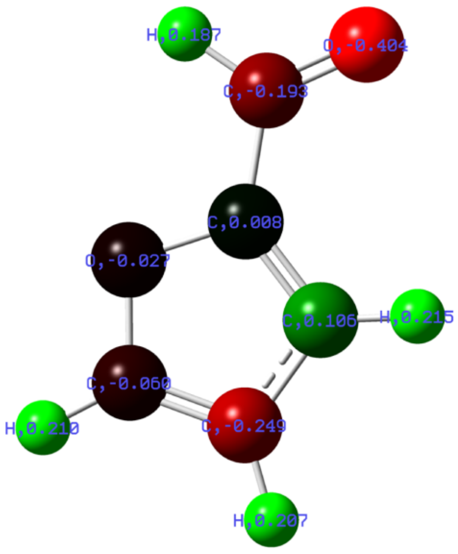

(b) The opted geometry of Furfural in 1-Octanol solvent

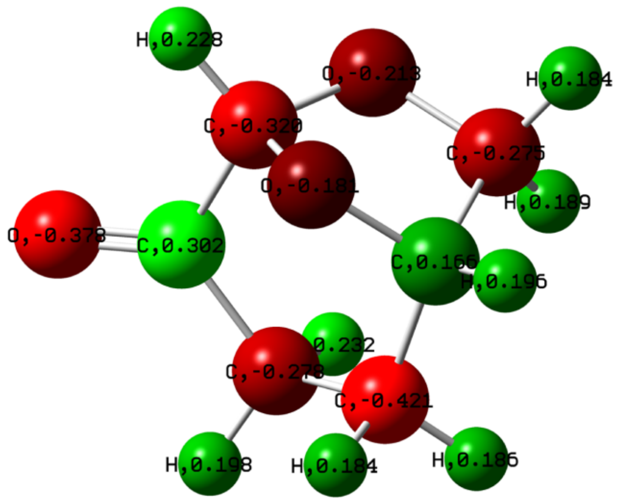

(c) The opted geometry of Dihydrolevoglucosenone in 1-Octanol solvent

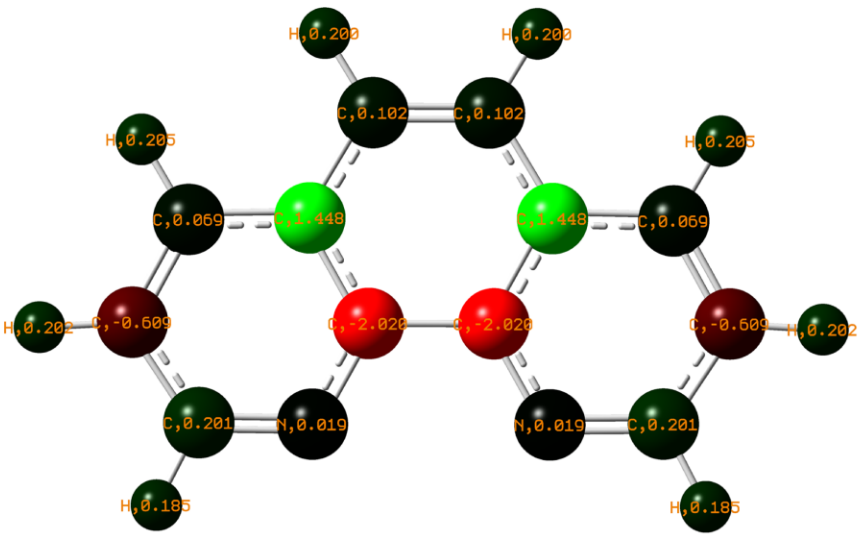

(d) The opted geometry of Phenanthroline in 1-Octanol solvent

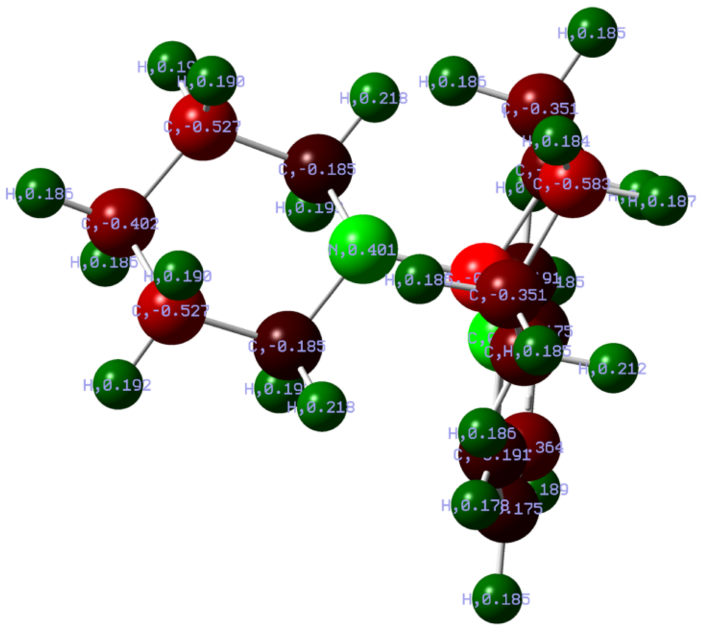

(e) The opted geometry of Phencyclidine in 1-Octanol solvent

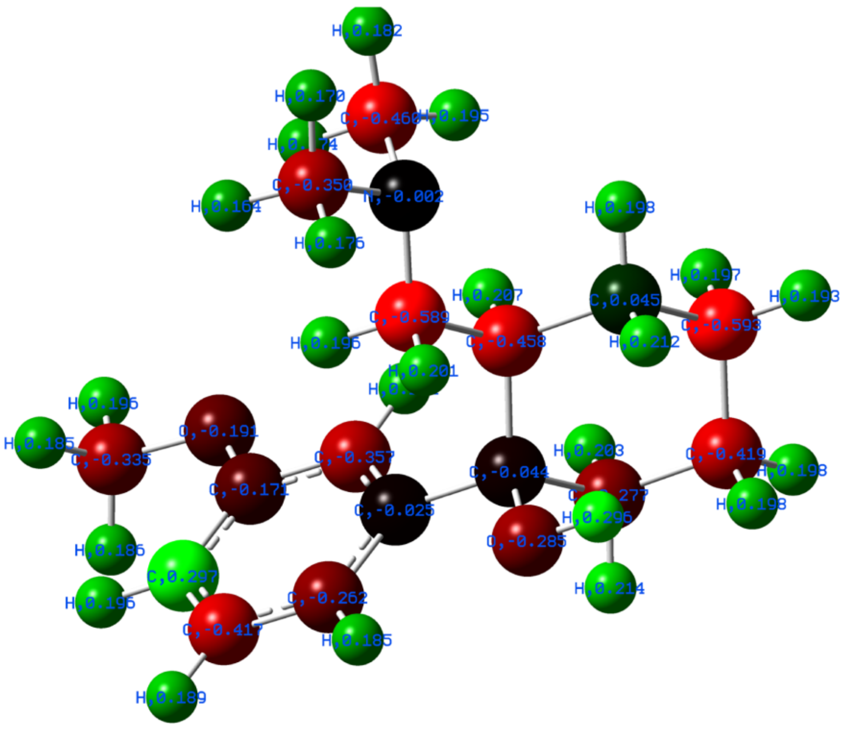

(f) The opted geometry of Tramadol in 1-Octanol solvent

Figure S3. Optimized geometries of the lowest energy conformers of the known compounds including **(a)** Phenol, **(b)** Furfural, **(c)** Dihydrolevoglucosenone, and some known drugs such as **(d)** Phenanthroline, **(e)** Phencyclidine, **(f)** Tramadol, **(g)** Cytarabine and **(h)** UNK4 at the M06-2X/6-311+G (d, p) level in 1-Octanol solvent (gauss view6) .

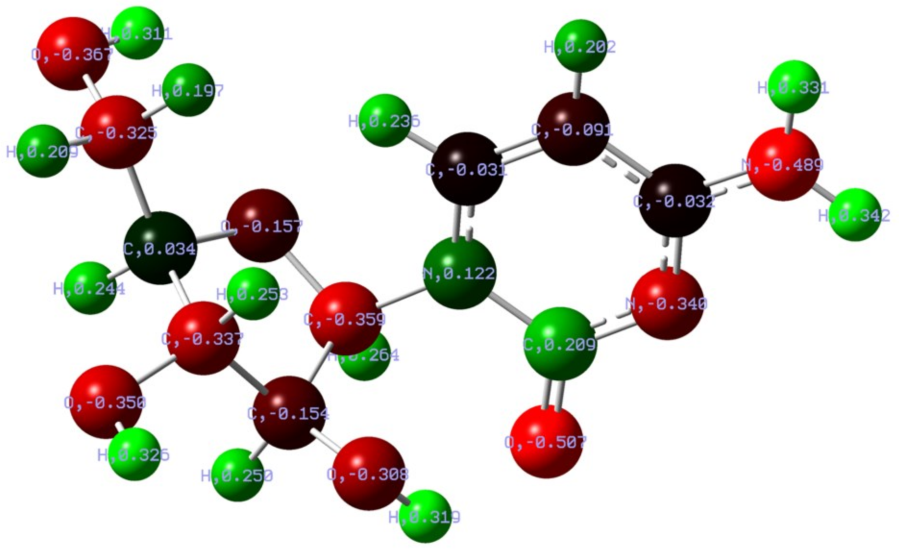

(g) The opted geometry of **Cytarabine** in 1-Octanol solvent

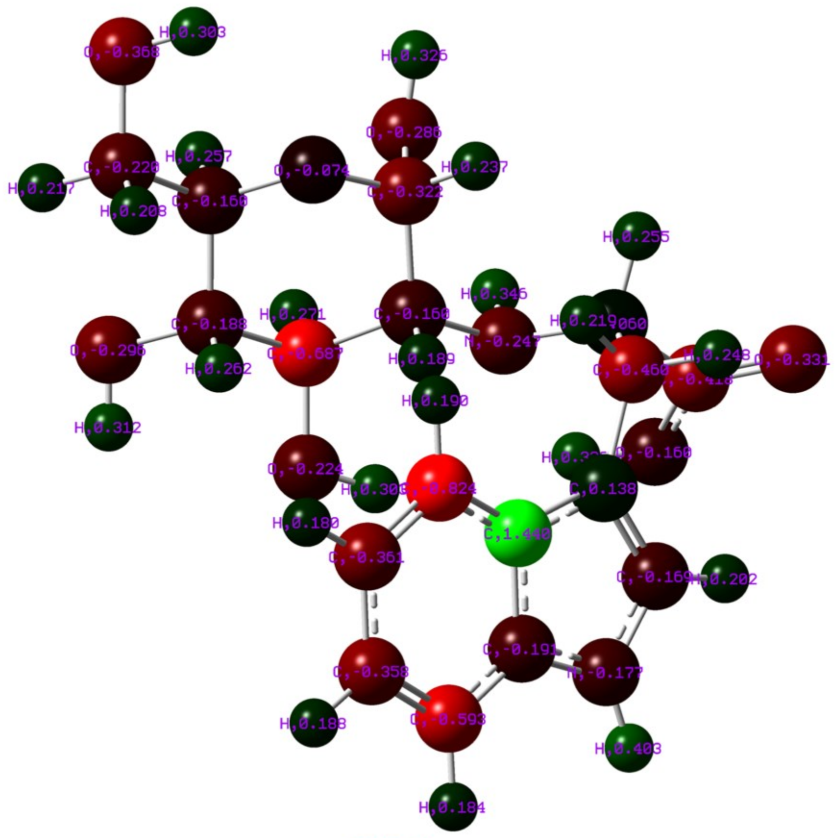

(h) The opted geometry of **UNK4** in 1-Octanol solvent

Figure S3. Optimized geometries of the lowest energy conformers of the known compounds including **(a)** Phenol, **(b)** Furfural, **(c)** Dihydrolevoglucosenone, and some known drugs such as **(d)** Phenanthroline, **(e)** Phencyclidine, **(f)** Tramadol, **(g)** Cytarabine and **(h)** UNK4 at the M06-2X/6-311+G (d, p) level in 1-Octanol solvent (gauss view6).

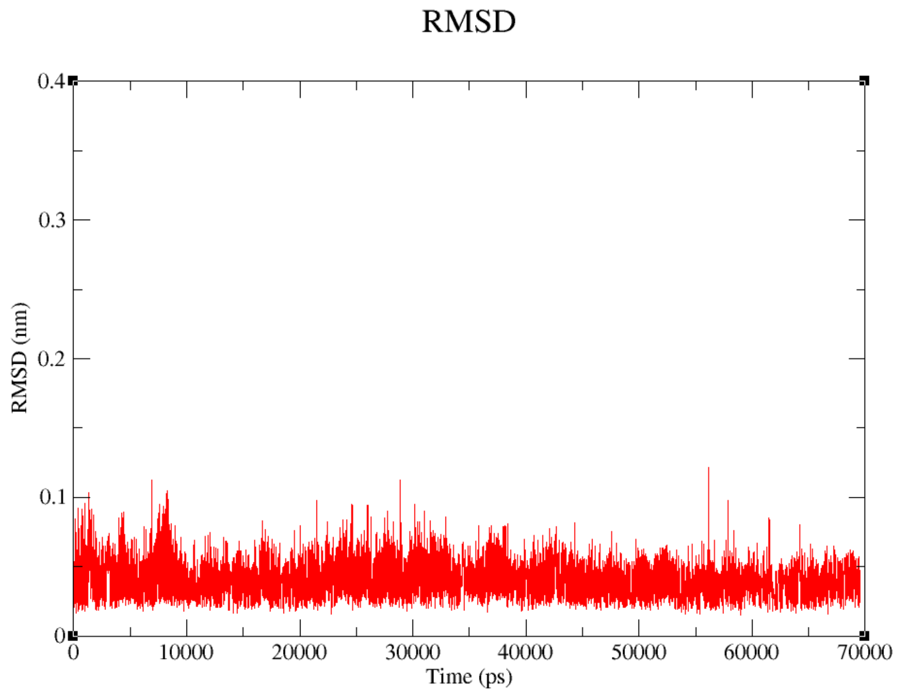

Figure S4. RMSD values of UNK4 during 70 ns of simulation.
