# Supplementary material for: Design of amino acid- and carbohydrate-based anticancer drugs to inhibit polymerase η
Source: Sci Rep. 2022 Nov 2;12:18461. doi: 10.1038/s41598-022-22810-z (PMC9630280; doi:10.1038/s41598-022-22810-z)
Supplement: Supplementary file 3 — Supplementary Information 3. [file 41598_2022_22810_MOESM3_ESM.pdf]

8 snapshots during the first 30 ns of MD simulation were taken for both CNP (Figure S5) and UNK4 (Figure S6) while were in complex with Polη and DNA. According to these figures (S5 and S6), UNK4 compared to CNP can have more prominent effect on complex of Polη and DNA. In these figures (S5and S6), Mg ions are shown in pink color and chloride ions are yellow. 2 strands are shown in cyan and blue colors. Solvent (water molecules) are displayed by h-bond model in red. Polη is shown by use of new cartoon style. Both UNK4 and CNP are shown by ball and stick model.

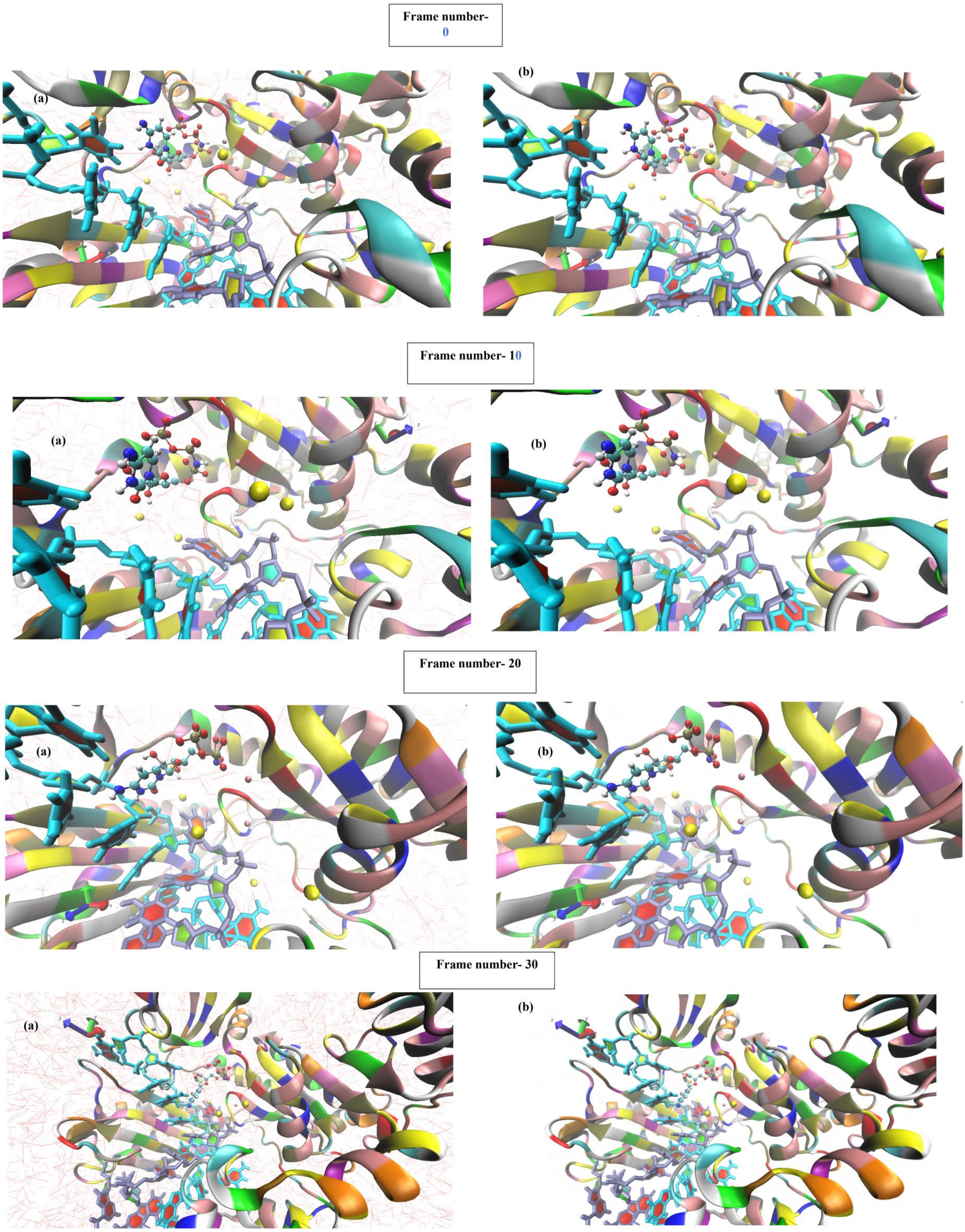

Figure S5. 8 snapshots during 30 ns of MD simulation for CNP while is in complex with Polη and DNA (VMD 1.9.3)
