# Supplementary figures and images for: Design of amino acid- and carbohydrate-based anticancer drugs to inhibit polymerase η
Source: Sci Rep. 2022 Nov 2;12:18461. doi: 10.1038/s41598-022-22810-z (PMC9630280; doi:10.1038/s41598-022-22810-z)

Frame number- 40

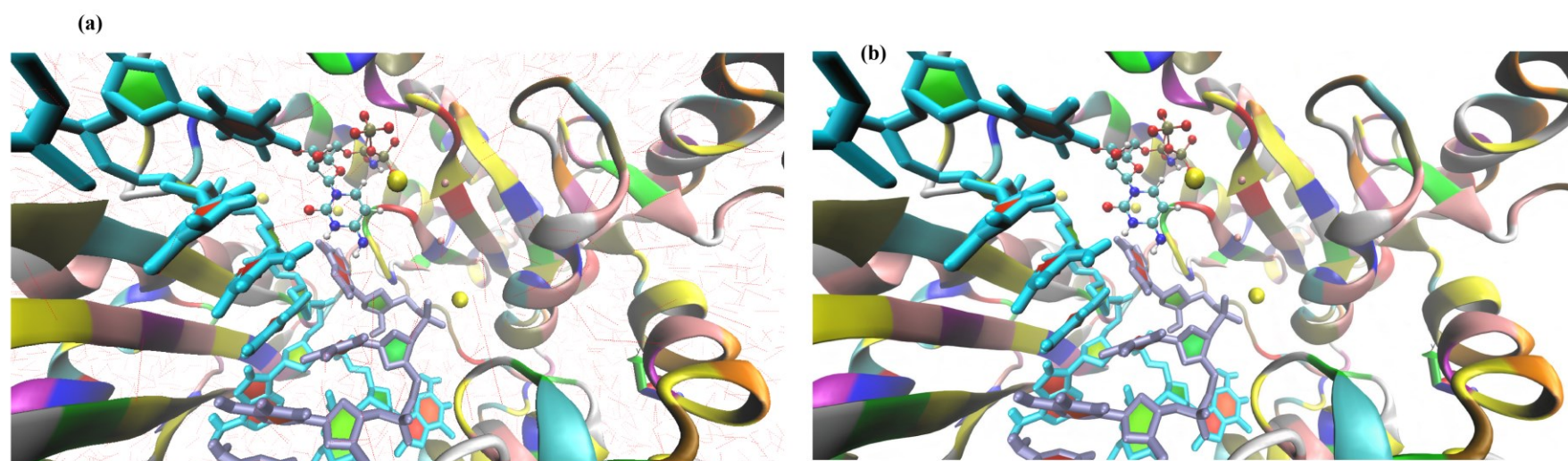

Frame number- 50

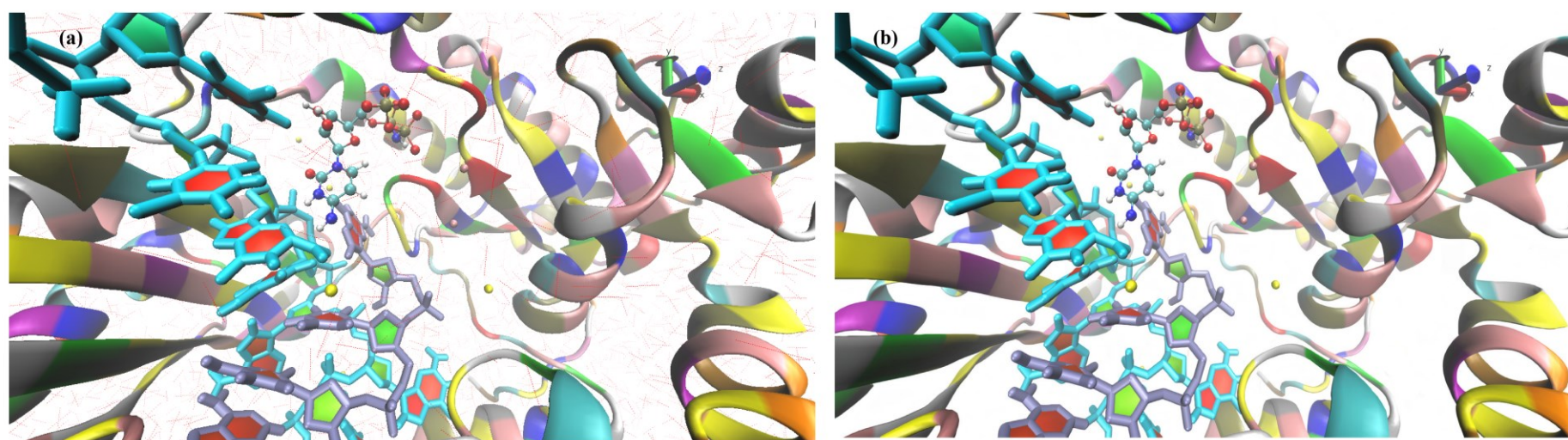

Frame number- 75

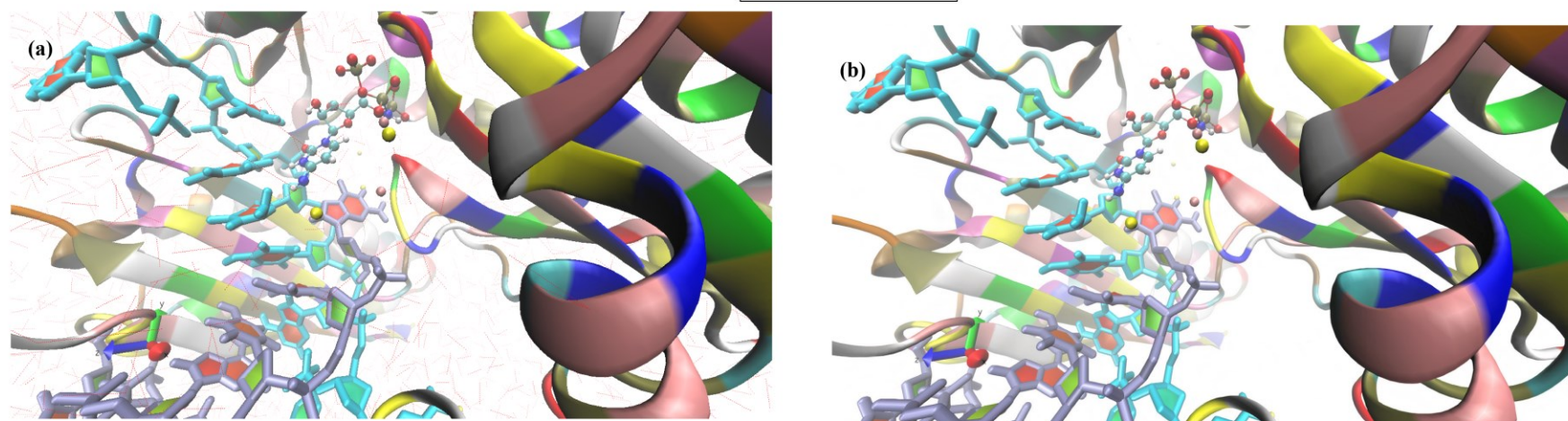

Frame number- 100

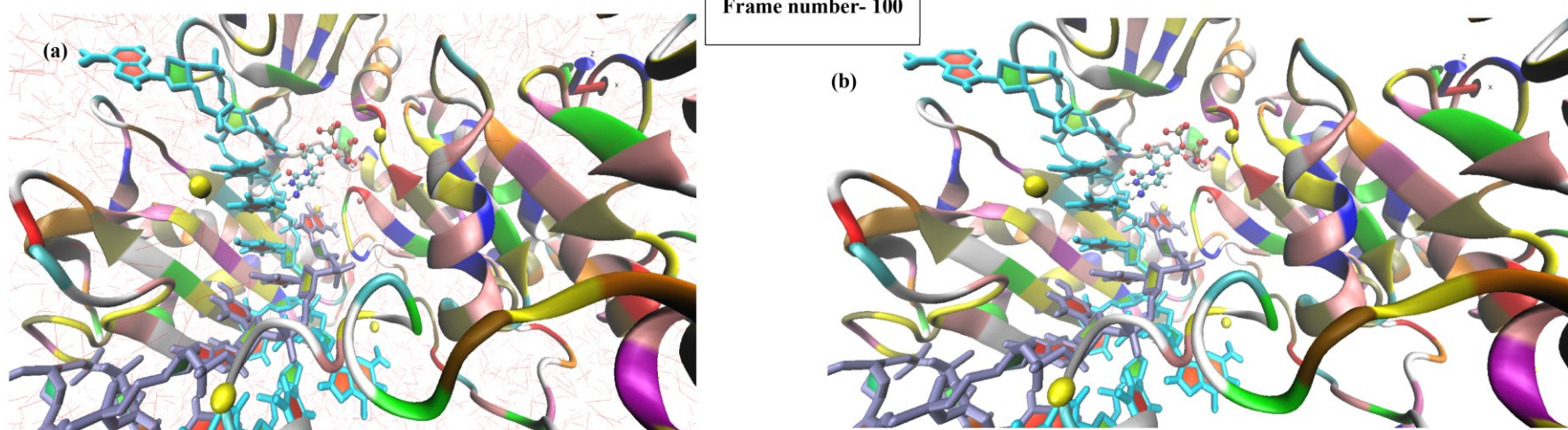

Figure S5. Continued (VMD 1.9.3).

Supplement: Supplementary file 4 — Supplementary Information 4. [file 41598_2022_22810_MOESM4_ESM.pdf]

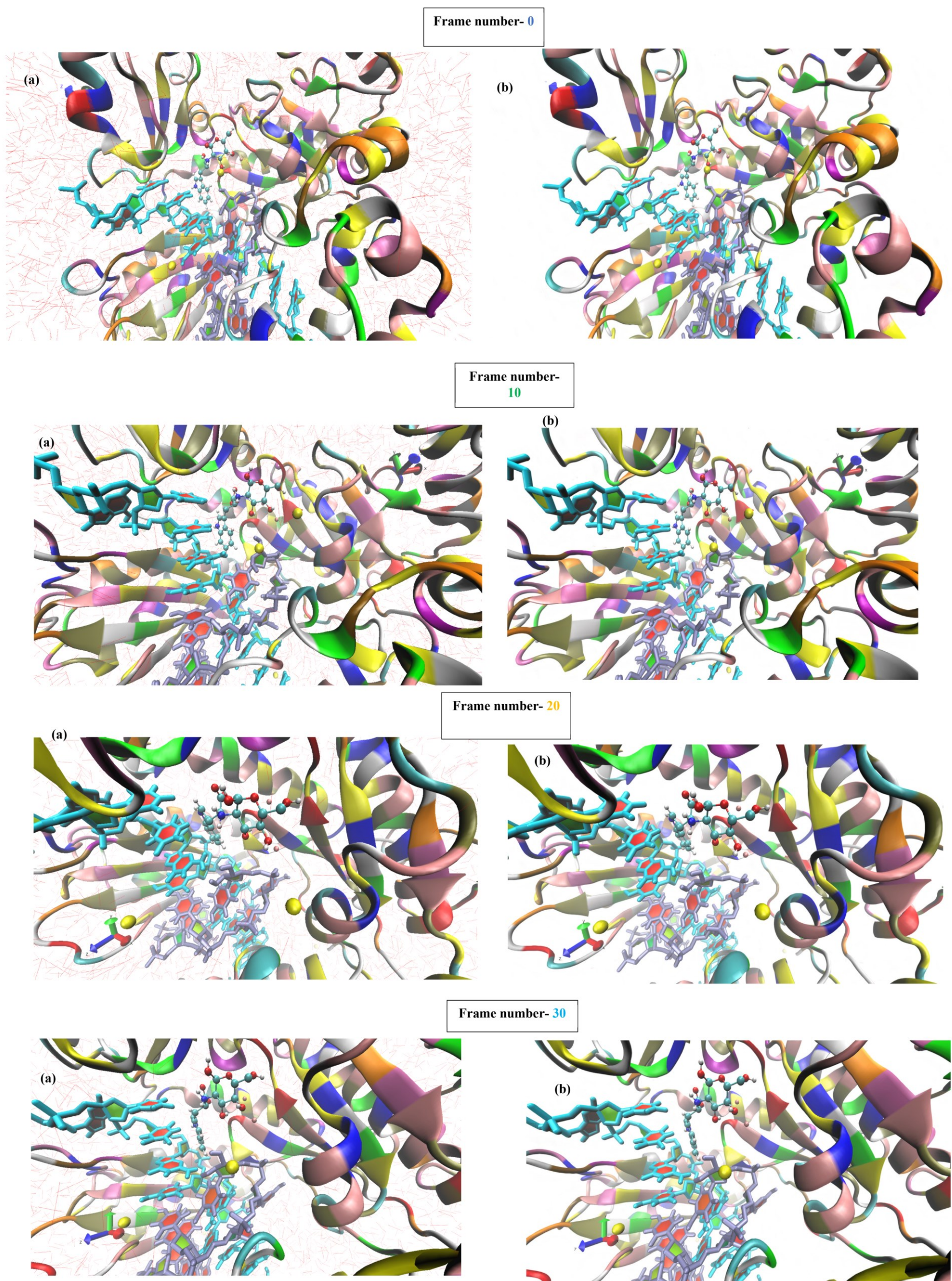

**Figure S6.** 8 snapshots during 30 ns of MD simulation for UNK4 while is in complex with Polη and DNA (VMD 1.9.3).

Supplement: Supplementary file 5 — Supplementary Information 5. [file 41598_2022_22810_MOESM5_ESM.pdf]
