# Supplementary material for: Design of amino acid- and carbohydrate-based anticancer drugs to inhibit polymerase η
Source: Sci Rep. 2022 Nov 2;12:18461. doi: 10.1038/s41598-022-22810-z (PMC9630280; doi:10.1038/s41598-022-22810-z)
Supplement: Supplementary file 6 — Supplementary Information 6. [file 41598_2022_22810_MOESM6_ESM.pdf]

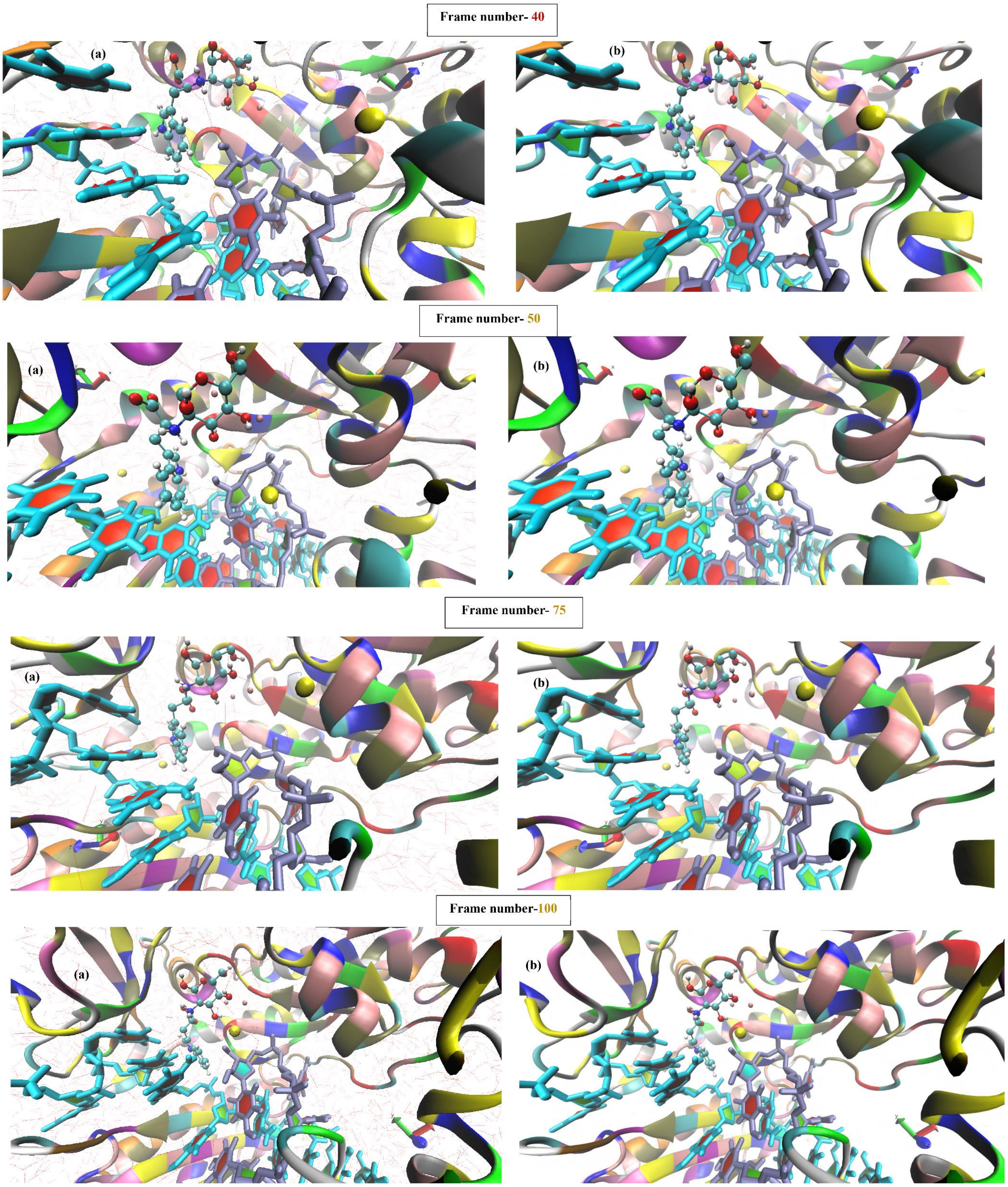

Figure S6. 8 snapshots during 30 ns of MD simulation for UNK4 while is in complex with Polη and DNA

-The FT-IR and NMR analyses of UNK4

The FT-IR spectrum of the resultant (UNK4) can be seen in Figure S7. The peaks observed in the broad signal at 3200-3500 cm^{-1} can be attributed to the D-fructose's OH (hydroxyl bonds); vibration peaks at 755.48 $cm^{-1}$and 855.48 cm^{-1} have been appeared due to =C-H. As can be seen in Figure S7, the frequencies related to the C– N stretching modes are perceptible in 1190 cm^{-1}. The stretching vibrations of carbonyl in COOH group manifests itself in 1700.82 cm^{-1}. FTIR spectra of samples were achieved using an ABB Bomem MB-100 FTIR spectrophotometer.

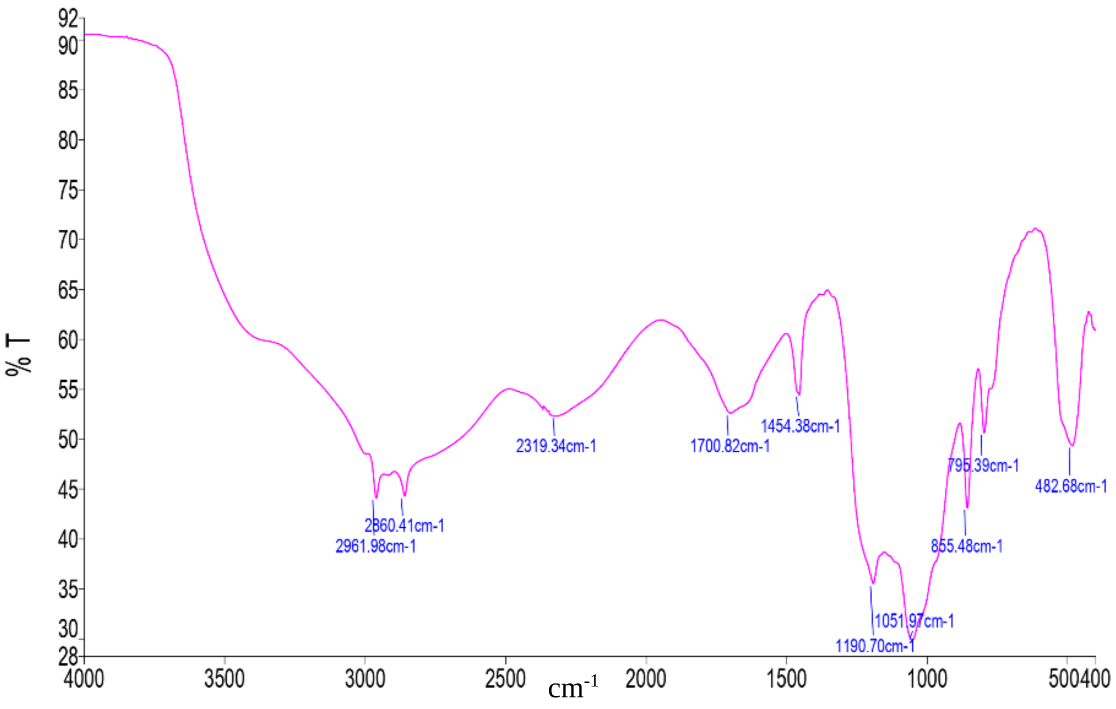

Figure S7. The FT-IR spectrum of the resultant (UNK4).

Table S2. ^{1}H NMR spectrum of UNK4 was recorded on a Bruker (Avance DRX-500) spectrometer using D_2O as solvent at room temperature.

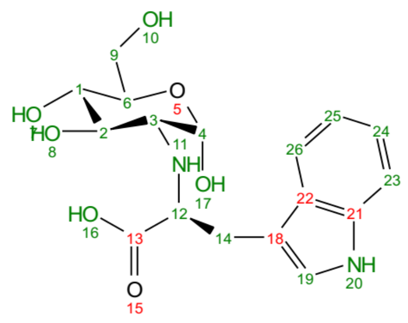

Atom	δ (ppm)	Number of hydrogen atoms	Min, Max (ppm)
1 C			
H	3.63	1	3.62, 3.64
2 C			
H	3.67	1	3.66, 3.68
3 C			
H	2.91	1	2.90, 2.92
4 C			
H	5	1	4.99, 5.01
5 O			
6 C			
H	3.93	1	3.92, 3.94
7 O			
H	4.76	1	4.75, 4.77
8 O			
H	4.64	1	4.63, 4.65
9 C			
H2	3.79	2	3.78, 3.80
10 O			
H	4.6	1	4.59, 4.61
11 N			
H	4.96	1	4.95, 4.97
12 C			
H	3.97	1	3.96, 3.98
13 C			
14 C			
H2	3.6	2	3.59, 3.61
15 O			
16 O			
H	16.3	1	16.29, 16.31
17 O			
H	2.3	1	2.29, 2.31
18 C			
19 C			
H	6.23	1	6.22, 6.24
20 N			
H	7.54 (hardly seen because of exchange with D_2O)	1	7.53, 7.55
21 C			
22 C			
23 C			
H	7.5	1	7.49, 7.51
24 C			
H	7.5	1	7.49, 7.51
25 C			
H	6.27	1	6.26, 6.28
26 C			
H	7.5	1	7.49, 7.51

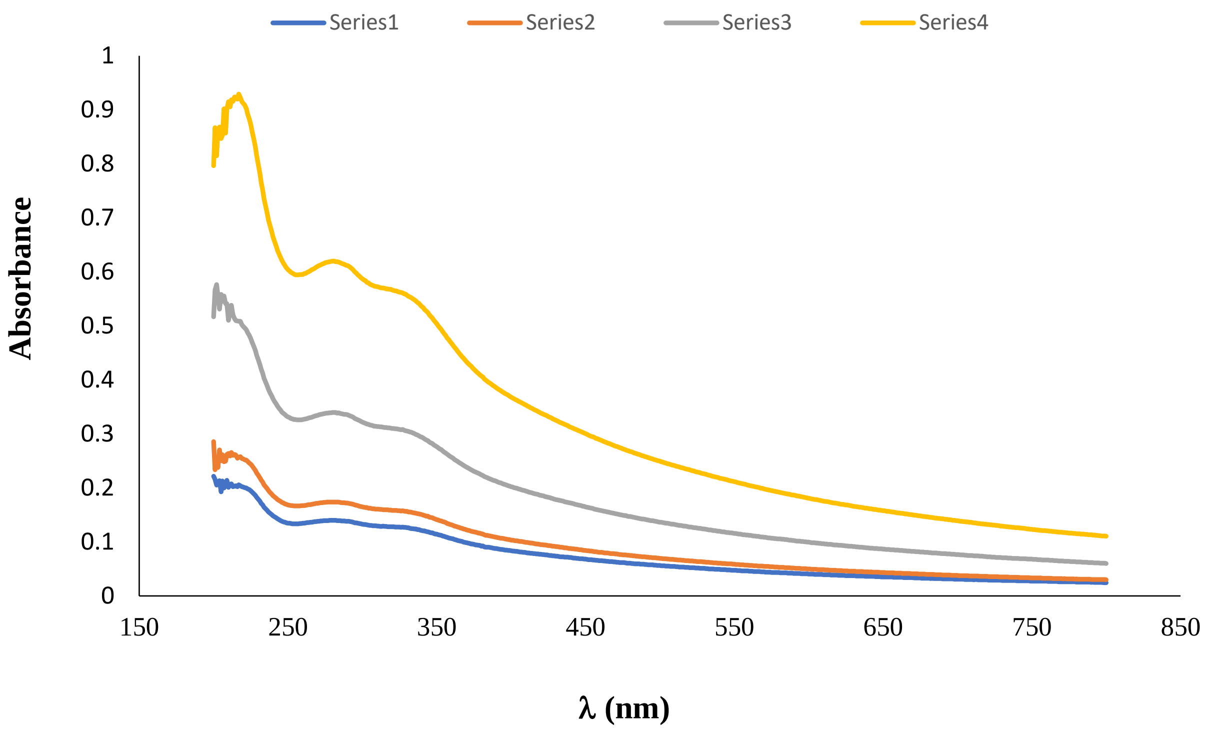

Figure S8. This figure shows the UV spectrum of the designed compound solutions (UNK4). In this figure, the yellow plot, grey plot, orange plot and blue plot represent the UV spectrums of solutions with concentrations of 0.0002964 M, 0.0003658 M, 0.0007316 M and 0.0014632 M, UV spectra were recorded on ultraviolet/visible spectrophotometer (PerkinElmer, Lambda 25).

Table S3. experimentally measured log P of UNK4

**$C_{int, aq}$ (M)**	**Absorbance after equilibration (A)**	**$C_{eq, aq}$ (M)**	K_{OW}	**log *P***
0.0002964 M	0.128907	0.000254647	0.163964	-0.78
0.0007316 M	0.31496	0.000714152	0.024432287	-1.61
0.0014632 M	0.58858	0.00139	0.001432	-1.52
